# The Quality of AI-Generated CABG Counseling: A Blinded Comparison of Two Language Models

**DOI:** 10.3390/jcm15103896

**Published:** 2026-05-19

**Authors:** Alper Özbakkaloğlu, Ömer Faruk Rahman, Ercan Keleş, Ahmet Daylan, Dağlar Cansu, Şahin Bozok

**Affiliations:** 1 Department of Cardiovascular Surgery, İzmir Bakırçay University, Menemen 35665, Turkey; alper.ozbakkaloglu@bakircay.edu.tr (A.Ö.); mevertra@yahoo.co.uk (E.K.); daglarcansu@icloud.com (D.C.); sahinboz@yahoo.com (Ş.B.); 2 Department of Cardiovascular Surgery, Ege University, Bornova 35040, Turkey

**Keywords:** coronary artery bypass grafting, large language models, artificial intelligence, patient education

## Abstract

**Objectives:** Coronary artery bypass grafting (CABG) remains a fundamental surgical treatment for advanced coronary artery disease. With the increasing use of large language models to obtain health information, patients are increasingly turning to these systems to understand surgical options. However, their performance in generating patient-oriented CABG information has not been sufficiently evaluated. Therefore, this study aimed to compare the responses generated by ChatGPT and DeepSeek-R1 to patient questions about CABG in terms of scientific accuracy, comprehensibility, and level of unnecessary detail. **Methods:** Forty patient-oriented questions were developed based on online sources and clinical experience. Responses were obtained from ChatGPT and DeepSeek under standardized conditions. A blinded panel of four cardiovascular surgeons evaluated the responses using a five-point Likert scale across three domains. Statistical analyses were performed using paired tests. **Results:** DeepSeek generated significantly longer responses than ChatGPT (212.88 ± 48.13 vs. 188.7 ± 50.34 words; *p* < 0.001). Accuracy scores were higher for DeepSeek (median 4.5 vs. 4.25; *p* = 0.004), whereas comprehensibility and unnecessary detail scores were similar between the models. Overall scores were high for both models (4.32 ± 0.28 vs. 4.27 ± 0.30; *p* = 0.34). **Conclusions:** The responses generated by both models were generally evaluated favorably by the expert panel, with only limited differences observed between them. DeepSeek demonstrated higher accuracy, whereas ChatGPT tended to produce shorter and more concise responses. However, given the variability observed at the individual-question level, these findings should be interpreted with caution. Large language models may support patient information delivery but should not be considered reliable stand-alone sources for clinical decision-making or patient counseling.

## 1. Introduction

Coronary artery bypass grafting (CABG) remains a fundamental surgical approach in the treatment of advanced coronary artery disease, with well-established efficacy and favorable long-term outcomes. In terms of long-term survival and the need for repeat revascularization, CABG continues to be regarded as the gold standard, particularly for patients with a complex coronary anatomy [1,2]. However, CABG has evolved beyond a single procedure performed exclusively through conventional median sternotomy; with the incorporation of minimally invasive techniques, it now encompasses a broad spectrum of surgical strategies and technical variations.

This diversity may create uncertainty for patients regarding the most appropriate surgical approach and can increase the demand for information during the preoperative period. Today, patients seeking to understand and compare surgical options are increasingly relying on not only physician consultation but also digital and interactive information sources. In recent years, the use of artificial intelligence-based conversational systems for obtaining health-related information has risen substantially [3,4].

In this context, evaluating the responses generated by large language models to patient-oriented questions about CABG techniques is of particular importance in terms of scientific accuracy and patient-level comprehensibility. Nevertheless, the performance of large language models in producing patient-focused content specific to cardiovascular surgery has not been sufficiently investigated. In the present study, responses generated by two different large language models to questions concerning conventional and alternative CABG techniques were systematically assessed by a blinded expert panel with regard to scientific accuracy, comprehensibility, and the level of unnecessary detail, and the performance of the models was compared.

While previous studies have evaluated the performance of large language models in patient counseling across various medical fields, those specifically focusing on patient-oriented coronary artery bypass grafting (CABG) counseling remain limited. The present study therefore provides a comparative evaluation of AI-generated responses to CABG-related patient questions, with a particular emphasis on surgical counseling and patient-centered communication.

## 2. Materials and Methods

### 2.1. Ethics Statement

This study was based exclusively on expert evaluation of artificial intelligence-generated content and did not involve human participants or patient data. Therefore, institutional review board approval was not required.

### 2.2. Question Development and Response Collection

The patient questions evaluated in this study were compiled to reflect the topics frequently raised by individuals seeking information about coronary artery bypass grafting surgery. Both online sources and clinical observations were used in the development of the question pool. To capture the information-seeking tendencies of users in Türkiye, titles, search suggestions, content descriptions, and user comments appearing in searches conducted on the Google search engine (Google LLC, Mountain View, CA, USA) and the video-sharing platform YouTube (YouTube LLC, a subsidiary of Google LLC, San Bruno, CA, USA) were systematically reviewed. Searches were performed using keywords such as “coronary bypass surgery,” “small incision bypass,” and “minimally invasive bypass.” Frequently asked questions identified on patient forums and hospital websites were also screened. In addition, questions commonly directed to physicians in routine clinical practice were recorded by the research team and incorporated into the evaluation process.

All collected questions were reviewed for similarity and redundancy. Following a simplification process, a final list of 40 questions was established. Questions 1–10 focused on standard coronary artery bypass grafting, questions 11–20 addressed minimally invasive coronary artery bypass grafting, and questions 21–40 compared various aspects of these two surgical approaches. During question development, themes such as preoperative and postoperative patient concerns, technical aspects of the surgical techniques, medication use, return to daily activities after discharge, and lifestyle modifications were considered. All questions were formulated in clear and simple Turkish to ensure comprehensibility for the general patient population in Türkiye.

The finalized questions were submitted to two different artificial intelligence-based conversational platforms on 10 August 2025 between 10:00 and 13:00, and responses were obtained. ChatGPT (OpenAI, San Francisco, CA, USA) and DeepSeek-R1 (DeepSeek AI, Hangzhou, China) were used in this study. Both platforms were accessed through publicly available web interfaces using free user accounts. Free versions were deliberately selected to reflect a widely accessible and standardized real-world user experience. ChatGPT responses were obtained via the web interface at https://chat.openai.com (accessed on 10 August 2025). This study was conducted after the public release of ChatGPT-5; however, when using the free web interface, detailed information about the exact model version is not provided.

DeepSeek responses were obtained via the web interface at https://deepseek.com (accessed on 10 August 2025). using a free user account. As indicated on the interface at the time of use, DeepSeek responses were generated using the DeepSeek R1 model.

During interactions with both models, no prior information, contextual framing, role assignment, or directive prompts that could influence the question-and-answer process were provided. Each question was submitted in a separate session by initiating a new conversation within the relevant interface. This approach ensured that each question was evaluated independently and prevented any influence of previous responses on subsequent outputs. Only the first response generated by each model was included in the analysis. No edits, abbreviations, or modifications were made to the original texts. Furthermore, no external intervention was applied to response length, and no limitations were imposed on word count or output length.

### 2.3. Expert Panel

All responses were generated in Turkish and evaluated in Turkish by the expert panel. The responses obtained from the artificial intelligence models were evaluated by a blinded expert panel consisting of four cardiovascular surgeons working at the same institution. The responses were organized into two separate anonymous booklets with model identity concealed; only the study designer knew which booklet corresponded to which model. To preserve the principle of blinding, the study designer did not participate in the evaluation process and was not included as a panelist. In addition, all responses were standardized in terms of formatting (Times New Roman, 12-point font), and any stylistic elements that could reveal model identity (such as emojis or platform-specific expressions) were removed to further enhance the blinding process.

The first booklet and the evaluation form were initially delivered to the panelists. A five-day interval was maintained between the first and second sessions to ensure that assessments were performed independently of any recall effects related to prior responses. Each response was scored using a five-point Likert scale under three categories. For each question, the final score was calculated as the arithmetic mean of the four panelists’ ratings. As the primary aim of this study was not to assess absolute agreement among experts but rather to reflect the average of independent clinical evaluations, inter-rater reliability was not considered a primary outcome; however, it was subsequently evaluated using the intraclass correlation coefficient (ICC) to provide additional information on scoring consistency.

To enhance consistency among panelists, the evaluation form included descriptions defining all score levels from 1 to 5 for each category. For scientific accuracy, a score of 1 indicated content containing major scientific or medical errors that could be misleading or potentially harmful; a score of 2 indicated responses with multiple important errors and low scientific reliability; a score of 3 indicated responses that were generally correct but contained certain deficiencies or minor errors; a score of 4 indicated content that was largely accurate with only minor deficiencies; and a score of 5 indicated content fully consistent with current guidelines and scientific evidence, without errors. Scientific accuracy was evaluated based on consistency with current clinical guidelines, established medical literature, and the clinical judgment of the expert panel.

For comprehensibility, a score of 1 indicated complex, jargon-heavy expressions with clear language problems; a score of 2 indicated responses that were difficult to understand, with a weak structure and unclear meaning; a score of 3 indicated texts in which the main message was understandable but some parts were confusing; a score of 4 indicated responses that were generally clear, with only minor issues in language or flow; and a score of 5 indicated fluent, logical responses that were easily understandable for the target audience.

For the level of unnecessary detail, a score of 1 indicated responses containing numerous irrelevant and distracting details that obscured the main message; a score of 2 indicated responses in which excessive or unnecessary explanations interfered with clarity; a score of 3 indicated texts that included some unnecessary information but preserved the core message; a score of 4 indicated responses with limited unnecessary information but overall focus; and a score of 5 indicated content that was concise, focused, and free of unnecessary information.

### 2.4. Statistical Analysis

Inter-rater reliability was assessed using the intraclass correlation coefficient (ICC [2, k]), based on a two-way random-effects model with absolute agreement and average measures, where “k” represents the number of raters.

All analyses were performed using IBM SPSS Statistics version 23 (IBM Corp., Armonk, NY, USA). The distribution of numerical variables was assessed using the Shapiro–Wilk test.

Individual Likert ratings were treated as ordinal variables, and non-parametric methods were used for the primary paired comparisons. In addition, scores obtained from multiple raters and evaluation domains were summarized using mean values to reflect overall expert assessment. This approach has been widely used in multi-rater evaluation studies and is considered acceptable when aggregated scores are analyzed across multiple observations and raters [5].

Differences between the paired response scores of the two models were compared using the paired-samples *t*-test for normally distributed data and the Wilcoxon signed-rank test for non-normally distributed data. The results are reported as the mean ± standard deviation for normally distributed variables and as median (minimum–maximum) values for non-normally distributed variables. As response lengths showed a normal distribution, comparisons between the two models were analyzed using the paired-samples *t*-test and are presented as the mean ± standard deviation. A *p*-value of less than 0.05 was considered statistically significant for all analyses. The interpretation of effect sizes was based on Cohen’s classification. Accordingly, for the Wilcoxon signed-rank test, r values of 0.1, 0.3, and 0.5 were considered to represent small, medium, and large effects, respectively. Similarly, for the paired-samples *t*-test, Cohen’s d values of 0.2, 0.5, and 0.8 were interpreted as small, medium, and large effects, respectively.

## 3. Results

The lengths of responses generated by ChatGPT and DeepSeek to the 40 patient questions included in this study are presented in Table 1. The mean response length was 188.7 ± 50.34 words for ChatGPT and 212.88 ± 48.13 words for DeepSeek. A statistically significant difference was observed between the two models in terms of response length (*p* < 0.001). DeepSeek produced significantly longer responses than ChatGPT.

In the expert evaluations, the comprehensibility score assigned by Reviewer 2 was lower for DeepSeek than for ChatGPT (median values of 4 and 5, respectively), and this difference was statistically significant (*p* = 0.02). For Reviewer 4, the accuracy score was higher for DeepSeek than for ChatGPT (median values of 4.5 and 4, respectively), and this difference was also statistically significant (*p* = 0.009). No statistically significant differences were identified between the two models in the remaining subdimension scores across the other reviewers.

When the overall subdimension scores were analyzed, accuracy was higher for DeepSeek (with a median value of 4.5, compared to 4.25 for ChatGPT), and this difference was statistically significant (*p* = 0.004). Comprehensibility scores were similar between the two models (with median values of 4.25 and 4.38 for DeepSeek and ChatGPT, respectively), and no statistically significant difference was observed (*p* = 0.66). Likewise, no significant difference was found in terms of unnecessary detail (median values of 4.25 and 4.25 for DeepSeek and ChatGPT, respectively; *p* = 0.48).

When the mean of the three categories was evaluated, it was found that DeepSeek demonstrated a slightly higher score than ChatGPT; however, this difference did not reach statistical significance (mean values of 4.32 ± 0.28 and 4.27 ± 0.30, respectively; *p* = 0.34). Detailed results including comparative analyses of reviewer scores and overall mean scores for both models are shown in Table 2.

Inter-rater agreement for both models was assessed using the intraclass correlation coefficient (ICC), based on a two-way mixed-effects model with a consistency definition and average measures. The ICC was 0.430 (95% CI: 0.074–0.672) for ChatGPT responses and 0.446 (95% CI: 0.101–0.681) for DeepSeek responses.

For single measurements, the ICC was 0.158 for ChatGPT and 0.168 forDeepSeek-R1.

The ICC values were statistically significant for both models (ChatGPT: *p* = 0.011; DeepSeek: *p* = 0.008).

## 4. Discussion

Our findings should be interpreted in the context of the existing literature evaluating the performance of large language models in patient counseling. In this study, the responses generated by ChatGPT and DeepSeek to patient questions regarding coronary artery bypass grafting were compared based on expert evaluations. The overall scores, calculated as the mean of three categories, exceeded 4 points for both large language models, indicating generally high and clinically acceptable performance. However, DeepSeek demonstrated significantly higher accuracy scores than ChatGPT. Despite these statistically significant differences, the high overall scores achieved by both models suggest that the observed differences may have limited clinical or practical relevance. In contrast, ChatGPT tended to produce shorter and more concise responses. In addition, similar scoring patterns were observed among the reviewers; although limited differences were noted in certain categories, the findings are generally consistent.

Similar findings have been reported in studies conducted across different clinical domains, indicating that large language models generally demonstrate comparable performance [6,7,8]. DeepSeek demonstrated higher accuracy scores and generated longer responses. This finding is consistent with that of previous studies reporting that DeepSeek tends to produce longer responses than ChatGPT in similar comparisons [9,10]. However, the fact that both models achieved mean accuracy scores above 4 suggests that the observed difference may have limited clinical significance. The assumption that longer responses are more accurate does not always hold true, and a direct relationship between response length and accuracy may not exist [11]. On the one hand, it has been reported that large language models may generate longer and more elaborate responses in situations of uncertainty; on the other hand, an increased text length may create a perception of greater completeness, leading users to evaluate such responses as more adequate [12]. However, longer responses do not necessarily result in more effective patient communication; excessive detail may increase cognitive load and hinder the understanding of the main message [13]. From this perspective, shorter and more concise responses may offer practical advantages. In line with this, the similar comprehensibility scores observed between the two models indicate that an increased response length does not necessarily translate into a better understanding.

Despite the overall high performance, both models showed lower mean scores for certain questions. The lowest mean score for ChatGPT was observed for the question “Does minimally invasive coronary bypass surgery require special equipment?”, whereas that for DeepSeek was observed for the question “In the long term, is the need for repeat stenting or a second bypass surgery lower with the minimally invasive approach compared with the conventional method?” This finding suggests that, although large language models generally produce satisfactory responses, their performance may be more variable at the level of individual questions [12,14].

Beyond aggregate scores, a qualitative examination of model responses revealed distinct patterns. In some cases, both models provided clear and understandable explanations that are consistent with current clinical knowledge (question 1; see Supplementary Material S1). Although no major factual errors were identified in the evaluated responses, not all answers were of the same quality, and certain limitations in content presentation were observed.

For example, in one response addressing antiplatelet therapy (question 3, ChatGPT response; see Supplementary Material S1), lifelong aspirin use was presented in a generalized manner. Although aspirin is commonly prescribed, antiplatelet strategies may vary depending on clinical factors, and treatment can be modified over time at the clinician’s discretion. Therefore, while not factually incorrect, such statements may be potentially misleading in a patient counseling context. These findings indicate that, despite generally high overall scores, model responses may vary in quality at the individual-question level.

Although no responses in our study were rated as clearly incorrect or at the lowest score level, those with relatively lower mean scores may still contain clinically relevant limitations. Previous studies have reported that large language models can occasionally generate inaccurate or misleading information, raising important concerns regarding their use in healthcare settings [15,16]. Therefore, these systems should not be considered reliable stand-alone sources of information and require clinician oversight.

### Limitations

This study has several important limitations. First, although it has an inherently multilevel data structure, including questions, raters, models, and domains, analyses were conducted using question-level paired comparisons and averaged rater scores in line with our primary objective. While this approach allows for a comparison of the overall performance of the two models, it does not allow for simultaneous modeling of variability at the question and rater levels. In addition, the limitations associated with the use of aggregated Likert scores and the low-to-moderate level of inter-rater agreement indicate the presence of variability in individual assessments. In addition, the ICC values for single measurements being lower than the average scores and the relatively wide confidence intervals further support the presence of inter-rater variability and limitations of the rating scale.

Furthermore, the relatively limited sample size, the small number of raters, and the concentration of scores in the upper range (ceiling effect) may have limited the applicability and stability of more complex multilevel models. For this reason, paired non-parametric analyses, which require fewer assumptions and provide a more conservative framework, were preferred.

Multiple related outcomes were evaluated across several domains within an exploratory framework, and no single primary endpoint was predefined. This may limit the interpretation of nominal *p*-values.

Moreover, outputs from large language models are sensitive to temporal and contextual factors and may vary accordingly. The use of a single response per question in this study may therefore limit the reproducibility of the findings.

Finally, the question set was developed by the research team based on online sources and clinical experience, which may introduce a degree of selection bias. In addition, the use of questions formulated in a single language may limit the generalizability of the findings, particularly given that large language models are known to exhibit variable performance across languages. Future studies using larger, more diverse, and potentially multicenter datasets may benefit from applying multilevel analytical approaches to better capture model- and rater-level effects.

## 5. Conclusions

Large language models have emerged as practical and accessible tools for answering patient questions in clinical contexts. In this study, the responses provided by ChatGPT and DeepSeek to patient questions regarding coronary artery bypass grafting received generally favorable evaluations from an expert panel, with only limited differences between the models. DeepSeek demonstrated higher accuracy, whereas ChatGPT tended to produce shorter and more concise responses.

These findings contribute to the existing literature on AI-generated patient counseling and provide insight into the strengths and limitations of large language models in CABG-related patient communication. However, considering the potential for errors and variability at the individual-question level, these models should not be regarded as reliable stand-alone sources of information. Therefore, their use in conjunction with clinician guidance is important for ensuring safe and effective patient counseling.

## Figures and Tables

**Table 1:** List of patient questions on coronary artery bypass surgery included in this study.

1	How many days will I stay in the hospital after coronary artery bypass surgery?
2	Is it necessary to use a chest corset after coronary artery bypass surgery?
3	Will I need to take blood thinning medication for life after coronary bypass surgery?
4	Do I need to wear compression stockings after coronary bypass surgery?
5	How long after coronary bypass surgery can I start driving?
6	When can I return to work after coronary bypass surgery?
7	How long after coronary bypass surgery can I resume sexual activity?
8	When can I travel by airplane after coronary bypass surgery?
9	Will I experience sleep problems after coronary bypass surgery?
10	Can I swim in the sea after coronary bypass surgery?
11	Can every coronary bypass surgery be performed using a minimally invasive approach?
12	In minimally invasive bypass surgery, can more than one vessel be treated?
13	Is minimally invasive coronary bypass surgery safe?
14	Is there a risk of converting to a conventional incision during minimally invasive bypass surgery?
15	Does minimally invasive coronary bypass surgery require special equipment?
16	During minimally invasive bypass surgery, is the leg vein also harvested using a closed technique?
17	How large will the scar be after minimally invasive coronary bypass surgery?
18	What are the advantages of minimally invasive bypass surgery?
19	I have previously undergone heart surgery. Can I have minimally invasive bypass surgery?
20	When can I resume sexual activity after minimally invasive coronary bypass surgery?
21	In elderly patients, is minimally invasive or conventional coronary bypass surgery safer?
22	For patients with diabetes, is minimally invasive or conventional coronary bypass surgery more appropriate?
23	In obese patients, is minimally invasive or conventional coronary bypass surgery safer?
24	For younger patients requiring bypass surgery, is the minimally invasive or conventional approach more suitable?
25	Can both conventional and minimally invasive coronary bypass surgery be performed without stopping the heart?
26	Is there a difference in operative time between minimally invasive and conventional coronary bypass surgery?
27	Is the risk of stroke lower with minimally invasive coronary bypass compared with the conventional approach?
28	Is there a difference in blood transfusion requirements between minimally invasive and conventional coronary bypass surgery?
29	Is the risk of wound infection lower with minimally invasive coronary bypass compared with the conventional method?
30	Is there a difference in intensive care unit stay between minimally invasive and conventional coronary bypass surgery?
31	Is there a difference in postoperative pain between minimally invasive and conventional coronary bypass surgery?
32	Is recovery from anesthesia easier after minimally invasive bypass compared with the conventional method?
33	Is the risk of postoperative depression or low mood different between minimally invasive and conventional coronary bypass surgery?
34	Is there a difference in the time to resume driving between minimally invasive and conventional coronary bypass surgery?
35	Is there a difference in the time to return to work between minimally invasive and conventional coronary bypass surgery?
36	Is there a difference in the time to resume sexual activity between minimally invasive and conventional coronary bypass surgery?
37	Is there a difference in the time to return to sports or heavy exercise between minimally invasive and conventional coronary bypass surgery?
38	Is there a difference in medication use after discharge between minimally invasive and conventional coronary bypass surgery?
39	After minimally invasive versus conventional bypass surgery, are follow up hospital visits more frequent?
40	In the long term, is the need for repeat stenting or a second bypass surgery lower with the minimally invasive approach compared with the conventional method?

**Table 2:** Comparison of reviewer scores for ChatGPT and DeepSeek across evaluation categories.

	ChatGPT	DeepSeek	Test Statistics	*p*	Effect Size
Accuracy (R1)	5 (2–5)	5 (3–5)	Z = −1.416	0.16	r = −0.224
Comprehensibility (R1)	4 (3–5)	4 (3–5)	Z = −0.159	0.87	r = −0.025
Unnecessary Detail (R1)	5 (3–5)	5 (3–5)	Z = −0.513	0.60	r = −0.081
Accuracy (R2)	5 (3–5)	5 (3–5)	Z = −1.213	0.22	r = −0.192
Comprehensibility (R2)	5 (3–5)	4 (3–5)	Z = −2.294	0.02	r = −0.363
Unnecessary Detail (R2)	5 (3–5)	4 (3–5)	Z = −0.853	0.39	r = −0.135
Accuracy (R3)	4 (3–5)	4 (3–5)	Z = −1.127	0.26	r = −0.178
Comprehensibility (R3)	4 (2–5)	4 (3–5)	Z = −1.091	0.27	r = −0.172
Unnecessary Detail (R3)	4 (3–5)	4 (3–5)	Z = −1.127	0.26	r = −0.178
Accuracy (R4)	4 (3–5)	4.5 (3–5)	Z = −2.599	0.009	r = −0.411
Comprehensibility (R4)	4 (3–5)	4 (3–5)	Z = 0.000	1.00	r = 0.000
Unnecessary Detail (R4)	4 (2–5)	4 (2–5)	Z = −0.354	0.72	r = −0.056
Accuracy (Overall)	4.25 (3.5–4.75)	4.5 (3.75–5)	Z = −2.847	0.004	r = −0.450
Comprehensibility (Overall)	4.38 (3–5)	4.25 (3.25–5)	Z = −0.439	0.66	r = −0.069
Unnecessary Detail (Overall)	4.25 (3–5)	4.25 (3.25–5)	Z = −0.711	0.48	r = −0.112
Mean of Three Categories (Overall)	4.27 ± 0.3	4.32 ± 0.28	t = −0.967	0.34	d = −0.153

R1–R4 indicate Reviewers 1–4. Values are presented as the median (min–max) unless otherwise stated. The mean of three categories is presented as the mean ± standard deviation.

## Data Availability

The data supporting the findings of this study are available within the article and its tables. Additional data may be obtained from the corresponding author upon reasonable request.

## Supplementary Materials

The following supporting information can be downloaded at https://www.mdpi.com/article/10.3390/jcm15103896/s1. Supplementary Material S1. Full Question Set and Model Responses.

## Abbreviations

The following abbreviations are used in this manuscript:

CABG	Coronary artery bypass grafting
AI	Artificial intelligence
LLM	Large language model
IRB	Institutional review board
R	Reviewer

## References

[1] Adeyemi A., Berman L., Staroselsky M., Cordero D., Hai O., Makaryus A.N., Zeltser R. (2025). Coronary Artery Bypass Grafting: A Review of Short- and Long-Term Outcomes. Int. J. Angiol..

[2] Mokhtassi S.S., Bulut H.I., Salmasi Y., Khoshbin E. (2025). Expert Review of the Strategies to Optimize Long-Term Outcomes After Coronary Artery Bypass Grafting. Rev. Cardiovasc. Med..

[3] Ayo-Ajibola O., Davis R.J., Lin M.E., Riddell J., Kravitz R.L. (2024). Characterizing the Adoption and Experiences of Users of Artificial Intelligence-Generated Health Information in the United States: Cross-Sectional Questionnaire Study. J. Med. Internet Res..

[4] Cetin H.K., Demir H.B., Demir T. (2025). ChatGPT’s Role in Coronary Artery Bypass Graft Information: A Critical Assessment. Sisli Etfal Hastan. Tip. Bul..

[5] Koo T.K., Li M.Y. (2016). A Guideline of Selecting and Reporting Intraclass Correlation Coefficients for Reliability Research. J. Chiropr. Med..

[6] Erkan M.H., Rahman Ö.F., Güner A., Ayyıldız F., Barbarus E. (2026). ChatGPT and Gemini in warfarin counseling. Croat. Med. J..

[7] Akçay O., Öztürk Ö., Acar T., Gürsoy S. (2025). Accuracy and Reliability of ChatGPT in Answering Patient Questions About Lung Cancer and Its Surgery: An Expert Panel Evaluation by Thoracic Surgeons. J. Cancer Educ..

[8] Rahman Ö.F., Özbakkaloğlu A., Arslangilay M., Daylan A., Keleş E., Bozkurt Ö.T., Bozok Ş. (2026). Expert evaluation of GPT-4o and Gemini responses to patient questions on carotid endarterectomy. Rev. Assoc. Med. Bras..

[9] Erkan M.H., Rahman Ö.F., Güner A., Ayyıldız F., Barbarus E. (2026). Comparative Analysis of Large Language Models in Hemodialysis Vascular Access: ChatGPT-5, Gemini-2.5, and DeepSeek-V3. Eur. Res. J..

[10] Zhang Y., Huang T., Liu C., Miller A.N., Yang M., Harris I.A., Sawaguchi T., Miclau T., Tian M., Chui C.S. (2026). Comparative evaluation of large language models for hip fracture-related patient questions: DeepSeek-V3-FW, Gemini 2.0 Flash, and ChatGPT-4.5. Digit. Health.

[11] Anh-Hoang D., Tran V., Nguyen L.M. (2025). Survey and analysis of hallucinations in large language models: Attribution to prompting strategies or model behavior. Front. Artif. Intell..

[12] Steyvers M., Tejeda H., Kumar A., Belem C., Karny S., Hu X., Mayer L.W., Smyth P. (2025). What large language models know and what people think they know. Nat. Mach. Intell..

[13] Baxter K.A., Sachdeva N., Baker S. (2025). The Application of Cognitive Load Theory to the Design of Health and Behavior Change Programs: Principles and Recommendations. Health Educ. Behav..

[14] Zhou L., Schellaert W., Martínez-Plumed F., Moros-Daval Y., Ferri C., Hernández-Orallo J. (2024). Larger and more instructable language models become less reliable. Nature.

[15] Roustan D., Bastardot F. (2025). The Clinicians’ Guide to Large Language Models: A General Perspective With a Focus on Hallucinations. Interact. J. Med. Res..

[16] Geracitano J., Anderson B., Coffel M., Rosenzweig M., Dorn S.D., Khairat S., Conklin J. (2025). The Accuracy of ChatGPT in Answering FAQs, Making Clinical Recommendations, and Categorizing Patient Symptoms: A Literature Review. Adv. Health Inf. Sci. Pract..

