# Supplementary material for: The Quality of AI-Generated CABG Counseling: A Blinded Comparison of Two Language Models
Source: J Clin Med. 2026 May 19;15(10):3896. doi: 10.3390/jcm15103896 (PMC13207662; doi:10.3390/jcm15103896)
Supplement: Supplementary file 1 [file jcm-15-03896-s001.zip › jcm-4281434-supplementary.pdf]

Supplementary Material S1. Responses of ChatGPT and DeepSeek to Patient-Oriented CABG Questions

1–ChatGPT Responses to Patient-Oriented CABG Questions

STANDARD CORONARY ARTERY BYPASS (10 QUESTIONS)

1. How many days will I stay in the hospital after coronary artery bypass surgery?

The length of hospital stay after coronary artery bypass surgery is generally between 5 and 7 days. However, this duration may vary depending on the complexity of the surgery, accompanying diseases (e.g., diabetes, kidney disease, or lung problems), postoperative complications, and the speed of recovery.

In general, the process is as follows:

1. Intensive care unit:

- Immediately after surgery, patients usually stay in the intensive care unit for 1–2 days.
- During this period, removal from the ventilator, heart rhythm monitoring, pain control, and wound care are performed.

2. Ward period:

- After leaving the intensive care unit, patients usually remain in the regular ward for 3–5 days.
- During this time, walking is initiated, chest tubes are removed, and nutrition and breathing exercises are organized.

3. Discharge:

- Patients are discharged if cardiac function, wound healing, and general condition are satisfactory.
- If complications such as arrhythmia, infection, or fluid accumulation develop, the hospital stay may be prolonged by several days.

In uncomplicated cases, returning home within 1 week is common. However, in some patients, especially elderly individuals or those with additional medical conditions, the duration may extend to 10–14 days.

If you would like, I can also explain step by step what precautions should be taken during the first month at home after discharge.

2. Is it necessary to use a chest corset after coronary artery bypass surgery?

Yes, the use of a chest corset (sternal corset or surgical vest) is recommended for many patients after coronary artery bypass surgery, and there are several important reasons for this.

1. Stabilizes the breastbone (sternum):
   During surgery, the sternum is divided and opened. The corset helps keep the bone stable during healing and supports proper fusion.

2. Reduces pain:
   It decreases the load placed on the chest during coughing, sneezing, getting up, or turning in bed, making movements safer and more comfortable.

3. Reduces the risk of complications:
   It lowers the risk of problems such as improper healing of the sternum (sternal separation), infection, or chest wall deformity.

4. Maintains posture:
   It helps prevent the shoulders from falling forward during recovery and supports proper posture.

How long should it be used?
It is generally recommended for 6–8 weeks (sometimes up to 12 weeks). The duration depends on bone healing speed, age, body weight, accompanying conditions such as diabetes, and the surgeon's protocol.

Usage tips:

- It is usually worn throughout the day and removed only while bathing.
- Some patients may also be advised to wear it during sleep, especially in the first weeks.
- The corset should not be excessively tight; it should support the chest without interfering with breathing.

If you would like, I can also explain step by step how to wear a chest corset correctly after coronary artery bypass surgery so that it can be used both comfortably and safely.

3. Will I need to take blood thinning medication for life after coronary bypass surgery?

Lifelong use of blood thinning (antithrombotic) medication is generally required after coronary artery bypass surgery (CABG), although the specific medication and duration depend on the individual situation.

Basic principles:

1. Aspirin

- Almost all bypass patients are advised to use low-dose aspirin (usually 75–100 mg/day) for life.
- The aim is to prevent clot formation both in the bypass grafts and in the native coronary arteries.

2. Dual therapy (aspirin plus another medication)

- If a coronary stent had been placed before surgery,
- Or if there is a high risk of graft occlusion after surgery (e.g., very small vessels, severe atherosclerosis, or previous early graft failure),
- A second medication such as clopidogrel, prasugrel, or ticagrelor may be added to aspirin for a limited period, usually 6–12 months.

3. Additional anticoagulation for other reasons

- If there are additional conditions such as arrhythmia (e.g., atrial fibrillation), a prosthetic valve, or deep vein thrombosis, lifelong treatment with warfarin or newer oral anticoagulants (NOACs) may be required.

In summary:

- Aspirin → lifelong in most patients.
- Additional medications → 3–12 months or longer depending on risk factors.
- The duration of treatment is determined by the cardiologist while also considering bleeding risk.

If you would like, I can also prepare a table showing the typical lifelong medication regimen after bypass surgery and how it changes under different circumstances.

4. Do I need to wear compression stockings after coronary bypass surgery?

The use of compression stockings after coronary artery bypass surgery is recommended in many patients, although it is not mandatory for everyone.

The main reason is that a vein graft, usually the saphenous vein, is often harvested from the leg during surgery.

Why are compression stockings recommended?

- They help prevent or reduce swelling in the leg from which the vein was harvested.
- They support circulation and may accelerate healing.
- They reduce the risk of deep vein thrombosis.

How long are they used?

- They are generally worn during the daytime for approximately 4–6 weeks after surgery and removed at night.
- If leg swelling persists or venous insufficiency is present, this period may be extended to 2–3 months.

Who may not need them?

- Patients whose graft was obtained from the arm or internal thoracic artery instead of the leg
- Patients without leg swelling
- Patients without venous disease or clotting risk

In summary, the doctor will determine whether compression stockings are necessary and for how long based on the graft source and the condition of the leg.

If you would like, I can also prepare a checklist showing in which situations compression stockings are mandatory and when they are optional after surgery.

5. How long after coronary bypass surgery can I start driving?

The recommended time to resume driving after coronary artery bypass surgery is generally around 4–6 weeks.

This period is necessary for healing of the breastbone (sternum) and for recovery of reflexes and concentration.

Why should driving not be resumed too early?

- In the first weeks after surgery, the sternum has not fully healed, and sudden braking or forceful steering movements may damage the bone.
- Pain medications may slow reflexes.
- Fatigue, decreased concentration, and mild weakness in arm movements may occur.

Doctors usually consider the following criteria:

- Absence of chest pain
- Ability to use the arms comfortably
- No fainting, dizziness, or sudden shortness of breath
- Approval during follow-up examination

In summary, most patients can begin driving after approximately 1 month once the surgeon or cardiologist confirms recovery during follow-up evaluation.

If you would like, I can also prepare a short home exercise program to help accelerate recovery during the first 4–6 weeks after surgery.

devam et

6. When can I return to work after coronary bypass surgery?

The timing of return to work after coronary artery bypass surgery depends on both the physical and psychological demands of the job and the patient's recovery speed. In general:

- Desk-based or light work (computer, office, administrative work, etc.)
    - Usually possible within 6–8 weeks.
    - However, concentration, fatigue, and healing of the sternum should be sufficiently improved.
- Moderately physical work (light lifting, standing work, service-related work, etc.)
    - Most commonly after 8–12 weeks.
- Heavy physical work (construction work, heavy lifting, jobs requiring intense physical effort)
    - Complete healing of the sternum and recovery of cardiac capacity are necessary, which generally takes 3–6 months.

Points to consider:

- Heavy lifting and pushing-pulling movements should be avoided until the sternum has fully healed (usually around 8 weeks).
- Exercise testing may help determine a safer timing for return to work.
- In some patients, fatigue, depression, or concentration difficulties may delay return to work.

If you would like, I can prepare a week-by-week return-to-work plan based on your occupation to make the process clearer. Is your job desk-based or physically demanding?

7. How long after coronary bypass surgery can I resume sexual activity?

The timing of resuming sexual activity after coronary artery bypass surgery is generally determined by the patient's ability to perform daily activities comfortably and by the recovery of the cardiovascular system.

General medical approach:

- On average, sexual activity may be resumed approximately 4–6 weeks after surgery if there are no complications and the patient feels well.
- This period is necessary for healing of the heart muscle, fusion of the sternum, and reduction of postoperative fatigue.
- If a patient can climb two flights of stairs without chest pain or shortness of breath, the heart can generally tolerate sexual activity.

Points to consider:

- During the first attempts, positions requiring less physical effort should be preferred, and sudden or intense exertion should be avoided.
- Until the sternum has healed, pressure from the partner's body weight directly on the chest should be avoided.
- If chest pain, severe palpitations, shortness of breath, or dizziness occur, the activity should be stopped and a physician should be consulted.

If you would like, I can also prepare a step-by-step guide for safely returning to sexual activity after bypass surgery, including both physical and psychological aspects.

8. When can I travel by airplane after coronary bypass surgery?

After coronary artery bypass surgery, it is generally recommended to wait 4–6 weeks before air travel.

The reasons include:

- The sternum and surgical incisions need adequate healing.
- Cabin pressure and reduced oxygen levels may place additional stress on the heart and lungs.
- Prolonged immobility during flights may increase the risk of clot formation, which can be particularly dangerous in the early postoperative period.

General practical approach:

- Short-distance flights (1–2 hours): usually after 4 weeks with physician approval.
- Long-distance flights (more than 4–6 hours): usually after 6–8 weeks, preferably with a medical report and medications available during travel.
- Approval from the cardiac surgeon or cardiologist should always be obtained before travel.

Additional precautions:

- Wear compression stockings.
- Maintain adequate hydration and avoid alcohol or excessive caffeine.
- Walk every 1–2 hours or perform leg exercises while seated.

If you would like, I can also prepare a “travel checklist” for cardiac patients before air travel to help minimize risks during the journey.

9. Will I experience sleep problems after coronary bypass surgery?

Yes, sleep problems are quite common after coronary artery bypass surgery. This is usually temporary and may result from several factors.

1. Physical causes

- Chest pain and tenderness: The incision line, healing sternum, or tension around the ribs may make comfortable sleeping difficult.
- Shortness of breath: The lungs may need time to regain full capacity, which may cause difficulty, especially while lying flat.
- Frequent urination: Diuretic medications used after surgery may interrupt sleep.

2. Psychological causes

- Anxiety and depression: Undergoing major heart surgery may lead to fears such as “what if it happens again,” making it difficult to fall asleep.
- Post-intensive care syndrome: Sleep disturbances experienced in the hospital may continue after returning home.

3. Medication side effects

- Some painkillers, blood thinners, or beta-blockers may affect sleep quality.

4. Limitations in sleeping position

- Sleeping on the side may be difficult during the first weeks, and changing from the usual sleeping position may make it harder to fall asleep.

Suggestions that may help recovery:

- Avoid heavy meals and caffeine 1–2 hours before bedtime.
- Practice light relaxation exercises or breathing exercises before sleep.
- Use pain medication as recommended by the physician before bedtime to control discomfort.
- Sleep with supportive pillows or in a slightly side-lying position to support the chest.
- Obtain sunlight exposure during the day and perform light walking exercises.

In most patients, sleep problems improve within 4–8 weeks after surgery. However, if symptoms persist longer than 2 months or are accompanied by severe fatigue, depression, or shortness of breath, medical evaluation is recommended.

If you would like, I can also prepare a list of the most comfortable sleeping positions during the first month after surgery, since sleeping position may significantly affect recovery.

10. Can I swim in the sea after coronary bypass surgery?

The timing of swimming in the sea after coronary artery bypass surgery depends on the type of surgery, the recovery process, and the physician's recommendations. In general, the following points are important:

1. The surgical area must heal:
   Entering the sea before complete healing of the sternum and surgical incisions may increase the risk of infection and wound opening. This usually requires approximately 6–8 weeks, although the duration may be longer in some patients.
2. Swimming and diving movements:
   Simply entering the water may be possible earlier, but swimming or diving increases pressure on the chest. Therefore, the first attempts are generally limited to light water exposure, while swimming and diving should begin only after physician approval.
3. Water hygiene:
   Open sea water and especially crowded public beaches may carry infection risk. Entering such environments before complete wound healing is risky.
4. Cardiac function and exercise capacity:
   The heart's ability to tolerate physical effort after bypass surgery is important. Swimming places additional stress on the cardiovascular system; therefore, participation in rehabilitation programs and physician evaluation are recommended beforehand.

In summary:

- Swimming in the sea is generally considered safe at least 6–8 weeks after surgery and only after physician approval.
- Patients should avoid entering the sea before complete wound healing and evaluation of cardiac condition.

If you would like, I can also prepare a guide regarding safe swimming and sea-related precautions after surgery.

MINIMALLY INVASIVE CORONARY ARTERY BYPASS (10 QUESTIONS)

11. Can every coronary bypass surgery be performed using a minimally invasive approach?

No, not every coronary artery bypass surgery can be performed using a minimally invasive approach. There are several important reasons for this:

1. Patient and vascular condition:

- In conventional coronary artery bypass surgery, the chest is opened through a long midline incision (sternotomy), which allows easy access to the heart and coronary vessels.
- Minimally invasive techniques, such as bypassing one or a few vessels through a small incision, may be suitable for some patients. However, they may not be feasible in patients with multivessel coronary artery disease, complex coronary occlusions, or previous cardiac surgeries.

2. Risks and technical limitations:

- Minimally invasive bypass surgery limits the surgeon’s field of view and access. Certain coronary arteries, especially those located on the posterior surface of the heart, may not be easily accessible through a small incision.
- In some patients, impaired cardiac function or accompanying medical conditions may make this approach riskier.

3. Balance between advantages and disadvantages:

- Small-incision surgery may shorten recovery time and reduce postoperative pain.
- However, complete and effective bypass of all diseased vessels cannot always be guaranteed, and in some cases conventional sternotomy may be preferred.

In summary, not every coronary artery bypass operation can be performed using a minimally invasive approach. The patient’s coronary anatomy, the number of blocked vessels, and overall health status are determining factors.

If you would like, I can also provide a table showing in which situations minimally invasive bypass surgery may be appropriate.

12. In minimally invasive bypass surgery, can more than one vessel be treated?

Yes, more than one coronary vessel can be treated during minimally invasive coronary artery bypass surgery (mini-thoracotomy or minimally invasive bypass surgery), although there are certain limitations.

1. Technical limitations:

- The incision is usually made on the side or front-side of the chest and is approximately 5–10 cm in length.
- Because the operative field is more limited, access is more difficult compared with conventional open-chest surgery performed through sternotomy.

2. Target vessels:

- In minimally invasive bypass surgery, the left anterior descending artery (LAD) is most commonly targeted, and occasionally one or two additional vessels may also be bypassed.
- If multiple bypass grafts are required, the surgeon may still attempt a minimally invasive approach, but operative time may be prolonged or some vessels may not be suitable for bypass through the limited incision.

3. Advantages and risks:

- Smaller incisions generally provide faster recovery, less bleeding, and less postoperative pain.
- However, if many vessels require treatment, conventional sternotomy may be safer and technically more feasible.

In summary, multiple-vessel bypass surgery can be performed through a minimally invasive approach, but the number and location of target vessels depend on the patient’s coronary anatomy and the surgeon’s experience.

If you would like, I can also illustrate and compare single-vessel and multivessel minimally invasive bypass procedures to clarify in which situations each approach is preferred.

13. Is minimally invasive coronary bypass surgery safe?

Minimally invasive coronary artery bypass surgery (often referred to as minimally invasive coronary artery bypass or mini-CABG) is currently considered a safe and effective option for selected patients. However, safety and success depend on the patient's condition and the surgeon's experience.

Advantages:

1. Smaller incisions:

- Instead of the long midline incision used in conventional surgery, a smaller incision of approximately 5–10 cm is generally used.

2. Faster recovery:

- Postoperative pain is usually less severe, hospital stay is shorter, and return to daily life is faster.

3. Lower risk of infection and bleeding:

- Compared with larger incisions, the risk of certain complications may be reduced.

4. Cosmetic advantage:

- The surgical scar is generally smaller.

Disadvantages and limitations:

1. Patient suitability:

- This approach is not appropriate for all patients with coronary artery disease. It may not be preferred in patients with extensive multivessel disease, urgent surgical indications, or severely impaired cardiac function.

2. Surgeon experience:

- Minimally invasive techniques are technically demanding and require experienced surgeons.

3. Limited vessel access:

- Some coronary arteries may not be easily reached through a small incision, and therefore conventional surgery may sometimes be necessary.

Safety:

- In appropriately selected patients and when performed by experienced surgeons, minimally invasive coronary bypass surgery is considered safe. Studies have shown complication rates similar to or lower than those of conventional open surgery.
- However, in patients requiring complex multivessel bypass surgery or with complicated cardiac conditions, the conventional approach may still be safer.

In summary, minimally invasive coronary bypass surgery is a safe and advantageous method when performed in the right patient by an experienced surgeon. Nevertheless, it may not be suitable for every patient, and evaluation by both a cardiologist and a cardiac surgeon is essential.

If you would like, I can also provide a table comparing situations in which minimally invasive surgery is preferred versus those in which conventional open surgery may be more appropriate.

14. Is there a risk of converting to a conventional incision during minimally invasive bypass surgery?

Yes, although the risk is generally low, conversion from a minimally invasive incision to a conventional sternotomy may occasionally be necessary during minimally invasive coronary artery bypass surgery.

1. Reasons for conversion:

- The surgical field is more limited during minimally invasive surgery. If the planned bypass grafts cannot be safely completed or unexpected technical difficulties arise, the surgeon may need to convert to a conventional sternotomy.
- Conversion may also become necessary in cases of bleeding, heart rhythm disturbances, or technical problems with graft connections.

2. Frequency:

- In experienced centers, the conversion rate is generally below 5%.
- This rate depends on both the surgeon's experience and the patient's coronary anatomy.

3. Impact on the patient:

- If conversion is required, patient safety remains the primary concern.
- Recovery time and postoperative discomfort may increase slightly, although long-term outcomes are generally similar.

In summary, the likelihood of conversion from a minimally invasive approach to a conventional incision is low but not completely absent. The surgeon evaluates this possibility before surgery based on coronary anatomy and risk factors.

If you would like, I can also prepare a table comparing the advantages and risks of minimally invasive versus conventional bypass surgery.

15. Does minimally invasive coronary bypass surgery require special equipment?

Yes, minimally invasive coronary artery bypass surgery requires specialized equipment and instruments because the operation is performed through a smaller and more limited surgical field compared with conventional open-chest surgery.

Examples of specialized equipment include:

1. Specialized surgical instruments:

- Longer and thinner forceps, scissors, and clamps designed for minimally invasive procedures
- Specialized suction and clamping devices adapted for limited-access surgery

2. Stabilization devices:

- Small stabilizers that reduce movement of the myocardium during surgery
- These devices may allow certain bypass procedures to be performed without stopping the heart

3. Camera and visualization systems:

- Some minimally invasive procedures require endoscopic cameras or fiber-optic lighting systems
- These systems improve visibility within the narrow operative field

4. Specialized retractors:

- Minimally invasive retractors are used to open the chest through a smaller incision

5. Hemostasis and suturing materials:

- Specialized needles and suturing materials designed for precise work in confined spaces

In summary, although minimally invasive bypass surgery is technically similar to standard bypass surgery, it requires specialized instruments and equipment to facilitate surgery through a limited operative field. Therefore, it may not be available in every hospital and requires both an experienced surgical team and appropriate technical infrastructure.

16. During minimally invasive bypass surgery, is the leg vein also harvested using a closed technique?

Yes, in many minimally invasive coronary artery bypass procedures, the leg vein can also be harvested using minimally invasive techniques. Instead of the traditional long incision along the leg, endoscopic or small-incision vein harvesting methods are commonly preferred.

With this technique:

- Small incisions of approximately 2–3 cm are made in the leg.
- The vein is removed using a small camera and specialized instruments.
- There is less tissue damage, recovery is faster, and the scar is generally smaller.

In summary, during minimally invasive coronary bypass surgery, not only the chest incision but also the vein harvesting procedure may be performed using minimally invasive methods. However, in some cases, conventional long-incision harvesting may still be necessary depending on the patient's vein anatomy or the surgeon's preference.

If you would like, I can also explain the endoscopic vein harvesting process step by step with illustrations.

17. How large will the scar be after minimally invasive coronary bypass surgery?

In minimally invasive coronary artery bypass surgery (MICS CABG), the incision is significantly smaller than the incision used in conventional open-chest surgery through sternotomy. The scar is generally approximately 5–10 cm in length.

Several important points should be noted:

- The incision is usually located on the left side of the chest near the breastbone.
- Since the operation is performed between the ribs, the sternum is generally not divided; instead, a small surgical "window" is created.

- The incision length may vary depending on the number of bypass grafts and the patient's anatomy. Single-vessel procedures may require shorter incisions, whereas procedures involving several vessels may require slightly longer ones.
- Over time, the scar usually fades and becomes less noticeable within several months.

If you would like, I can also provide an illustration comparing the scar size of minimally invasive surgery with that of conventional open surgery.

18. What are the advantages of minimally invasive bypass surgery?

Minimally invasive coronary artery bypass surgery offers several important advantages.

1. Smaller scar

- Instead of the 10–15 cm incision typically used in conventional surgery, a smaller incision of approximately 5–8 cm is usually sufficient.
- This may provide cosmetic benefits and result in a less visible scar.

2. Lower bleeding risk

- Compared with conventional open surgery, there is generally less tissue and vascular trauma, which may reduce bleeding risk.

3. Faster recovery

- Because the chest is opened less extensively, patients may recover more quickly and return to daily activities sooner.

4. Less postoperative pain

- Since the sternum is usually not fully divided, postoperative pain may be less severe.

5. Shorter hospital stay

- Hospitalization is often shorter, commonly around 3–5 days.

6. Lower infection risk

- Smaller incisions and reduced tissue trauma may decrease the risk of infection.

7. Earlier return to daily activities

- Patients may resume daily activities, work, and light exercise sooner.

8. Fewer complications in selected patients

- In patients with diabetes or obesity, minimally invasive approaches may reduce the risk of sternal wound infection or delayed wound healing.

However, the suitability of this approach depends on the patient's coronary anatomy, cardiac function, and overall health condition. In some situations, conventional open surgery may still be safer.

If you would like, I can also prepare a table comparing minimally invasive and conventional bypass surgery to make the differences clearer.

19. I have previously undergone heart surgery. Can I have minimally invasive coronary bypass surgery?

If you have previously undergone heart surgery, the possibility of minimally invasive coronary bypass surgery depends on your individual condition and the type of previous operation.

1. Type of previous surgery:

- If the previous operation was performed through a conventional sternotomy, scar tissue and adhesions may have developed around the breastbone and heart. These adhesions may make minimally invasive surgery more difficult or may limit access to certain coronary arteries.
- If the previous surgery was also performed through a small incision, another minimally invasive procedure may still be possible, although careful evaluation is necessary.

2. Coronary anatomy and number of diseased vessels:

- Minimally invasive approaches are often more suitable for one- or two-vessel bypass procedures. Patients requiring extensive multivessel bypass surgery may still require conventional open surgery.

3. Surgeon experience and technical resources:

- Minimally invasive cardiac surgery requires specialized equipment and significant surgical expertise. The experience of the surgeon with such procedures is important.

4. Risks:

- Adhesions from previous surgery may increase the risk of complications during minimally invasive surgery.
- In some cases, surgery may begin as minimally invasive but require conversion to a conventional sternotomy during the operation.

In summary, a previous heart surgery does not completely prevent minimally invasive bypass surgery, but suitability must be evaluated individually. The cardiac surgeon usually reviews imaging studies such as CT scans, coronary angiography, and previous operative reports before making a decision.

If you would like, I can also prepare a table outlining the criteria used to evaluate minimally invasive bypass surgery in patients with previous cardiac operations.

COMPARISON BETWEEN CONVENTIONAL AND MINIMALLY INVASIVE CORONARY BYPASS SURGERY (20 QUESTIONS)

20. When can I resume sexual activity after minimally invasive coronary bypass surgery?

The timing of resuming sexual activity after minimally invasive coronary artery bypass surgery depends on overall health status, the type of surgery performed, and the speed of recovery. Recovery is often somewhat faster after minimally invasive procedures, although caution is still necessary.

General recommendations:

1. Wait for the initial follow-up evaluation:

- It is important to attend follow-up examinations approximately 4–6 weeks after surgery. During these visits, the condition of the heart and chest is evaluated.

2. Listen to your body:

- If chest pain, shortness of breath, severe fatigue, or palpitations occur, sexual activity should be postponed and medical advice should be sought.

3. Resume gradually:

- Initial sexual activity should be gentle and gradual, with intensity increased slowly over time if tolerated.

4. Follow physician recommendations:

- Patients with heart failure, arrhythmias, or extensive multivessel surgery should carefully follow their physician's specific recommendations.

In general, most patients can safely resume sexual activity approximately 4–6 weeks after minimally invasive coronary bypass surgery, although recovery time varies between individuals.

If you would like, I can also prepare a step-by-step guide for safely resuming sexual activity after surgery.

21. In elderly patients, is minimally invasive or conventional coronary bypass surgery safer?

The choice between minimally invasive coronary artery bypass surgery and conventional sternotomy in elderly patients depends on several factors, including the surgeon's experience, the patient's overall condition, accompanying diseases, and coronary anatomy.

1. Conventional sternotomy approach

- The breastbone is fully divided, allowing complete access to all coronary arteries.
- Operative duration may be similar or slightly longer, but vessel exposure is generally easier.
- In elderly patients, bone healing problems and infection risk may be increased.
- Risks of bleeding, infection, and respiratory complications may be somewhat higher.
- This approach is often preferred for multivessel bypass procedures or complex coronary anatomy.

2. Minimally invasive approach (mini-sternotomy or lateral thoracotomy)

- The breastbone is not fully divided, and the incision is usually approximately 5–10 cm in length.
- Reduced tissue trauma may lead to less pain, earlier mobilization, and shorter hospitalization.
- In elderly patients, the risks of respiratory complications and wound infection may be lower.
- However, the technique is technically more demanding, and access to all coronary arteries may not always be possible.
- Some studies have shown that in carefully selected elderly patients, minimally invasive surgery may provide outcomes comparable to those of conventional surgery.

3. General clinical considerations

- In elderly patients with significant comorbidities, minimally invasive approaches may reduce complication risk when patient selection is appropriate.
- In patients with extensive multivessel disease or challenging anatomy, the conventional approach may still be safer and preferable.

- The surgeon’s experience is one of the most important factors influencing outcomes in minimally invasive surgery.

In summary, minimally invasive approaches may safely reduce the risks of infection, bleeding, and respiratory complications in carefully selected elderly patients. However, conventional surgery remains the standard approach in patients with complex coronary anatomy or extensive disease. The decision should be individualized according to patient characteristics and surgical expertise.

22. For patients with diabetes, is minimally invasive or conventional coronary bypass surgery more appropriate?

When planning coronary artery bypass surgery in patients with diabetes, several important factors should be considered when deciding between minimally invasive surgery and conventional open-heart surgery.

1. Wound healing and infection risk

- Diabetes may delay wound healing and increase the risk of infection.
- Because minimally invasive procedures use smaller incisions, the risk of wound infection is generally lower.
- Conventional sternotomy involves a larger incision and division of the breastbone, which may increase the risk of wound infection, poor bone healing, or wound separation in diabetic patients.

2. Operative complexity and number of bypass grafts

- Minimally invasive approaches are generally more suitable for one- or two-vessel bypass procedures.
- If extensive multivessel bypass surgery or complex coronary anatomy is present, the conventional approach may be safer.

3. Hospital stay and recovery time

- Minimally invasive surgery is usually associated with shorter hospitalization and faster recovery.
- Faster recovery may be advantageous in diabetic patients because prolonged hospitalization can make blood glucose control more difficult.

4. Blood glucose management

- In diabetic patients, careful blood glucose control before and after surgery is essential regardless of the surgical technique used.
- Poor glucose control increases the risk of infection and complications; therefore, glycemic management is as important as the surgical approach itself.

In summary:

- Minimally invasive surgery may provide advantages in diabetic patients by reducing wound-related complications and accelerating recovery.
- However, in patients with extensive coronary disease or anatomically complex lesions, the conventional approach may still be safer.

- The final decision depends on the patient's coronary anatomy, diabetes control, and overall health condition as assessed by the cardiac surgeon.

23. In obese patients, is minimally invasive or conventional coronary bypass surgery safer?

In obese patients, there are several important differences between minimally invasive coronary artery bypass surgery and conventional sternotomy with regard to safety. The most appropriate approach depends on the patient's general condition and coronary anatomy.

1. Conventional coronary artery bypass surgery (full sternotomy)

Advantages:

- Provides full access to the heart and coronary arteries
- Facilitates multivessel bypass surgery
- May be safer in patients with complex anatomy

Disadvantages in obese patients:

- Because the breastbone is fully divided, the risk of wound infection may be higher, particularly deep sternal wound infection
- Wound healing may be prolonged
- Mechanical ventilation and hospitalization may be longer

2. Minimally invasive coronary bypass surgery

Advantages:

- Because the breastbone is not fully opened, the risk of infection may be lower
- The incision is smaller, and recovery and hospitalization are generally shorter
- Blood loss and the need for blood transfusion may be reduced

Disadvantages:

- Surgical access is more limited, and some coronary arteries may be difficult to reach
- Operative time may be slightly longer
- This approach may not be suitable for all patients, particularly those requiring extensive multivessel bypass surgery

3. Special considerations in obese patients

- Obesity increases the risk of sternal wound infection, which may make minimally invasive approaches advantageous.
- Because the chest wall is less extensively disrupted, postoperative respiratory function may be better preserved with minimally invasive surgery.
- However, if coronary anatomy is unfavorable, minimally invasive surgery may not be technically safe.

In summary:

- In obese patients, minimally invasive surgery may offer advantages regarding infection risk and recovery time.
- However, in patients with complex coronary anatomy or extensive disease, conventional surgery may still be safer.
- The final decision should be based on the surgeon's experience, coronary anatomy, and the patient's overall condition.

24. For younger patients requiring bypass surgery, is the minimally invasive or conventional approach more suitable?

In younger patients who require coronary artery bypass surgery, the choice between minimally invasive surgery and conventional open-heart surgery depends on the patient's condition, the extent of coronary artery disease, and the surgeon's experience.

1. Minimally invasive approach

Advantages:

- Faster recovery because the breastbone is usually not fully divided
- Less postoperative pain and a smaller scar
- Shorter hospitalization
- Potentially reduced blood loss and infection risk

Disadvantages:

- Complete revascularization may not always be possible, especially in extensive multivessel disease
- Surgical experience is critically important, and the procedure may not be available in every center
- Access may be more limited in emergency situations

2. Conventional sternotomy approach

Advantages:

- Easier access to multiple coronary arteries
- Safer in patients with complex anatomy or urgent surgical needs
- Long-term outcomes are well established because this has been the standard approach for many years

Disadvantages:

- Recovery is generally slower because the breastbone is divided
- Postoperative pain and scar size are usually greater
- Bleeding and infection risks may be somewhat higher compared with minimally invasive surgery

3. Special considerations in younger patients

- Younger patients with good overall health may particularly benefit from the faster recovery and quality-of-life advantages associated with minimally invasive surgery.
- However, if coronary disease is extensive or anatomically complex, the conventional approach may be safer.
- If complete bypass surgery can be achieved minimally invasively and the surgeon is experienced, this approach may often be preferred.

In summary, minimally invasive surgery may be advantageous in carefully selected younger patients, whereas conventional surgery remains a reliable standard in patients with complex or extensive coronary disease.

25. Can both conventional and minimally invasive coronary bypass surgery be performed without stopping the heart?

Yes, both conventional coronary artery bypass surgery performed through sternotomy and minimally invasive coronary bypass surgery can be performed without stopping the heart. This technique is known as off-pump coronary artery bypass surgery (OPCAB).

1. Conventional coronary bypass surgery

- Traditional bypass surgery is often performed using cardiopulmonary bypass with the heart temporarily stopped.
- However, off-pump surgery may also be performed, particularly in patients in whom the use of the heart-lung machine may increase risk.
- Potential advantages include reduced inflammatory response and fewer complications related to cardiopulmonary bypass.
- However, surgical exposure and access to certain coronary arteries may be more technically challenging.

2. Minimally invasive coronary bypass surgery

- Minimally invasive procedures are often performed without stopping the heart because the limited surgical exposure makes use of the heart-lung machine more technically difficult.
- Surgeons typically use specialized stabilizers and operative techniques to perform bypass grafting on the beating heart.
- Potential advantages include reduced surgical trauma, faster recovery, and shorter hospitalization.
- However, multivessel procedures may be technically more difficult and outcomes are more dependent on surgical experience.

In summary:

- Both conventional and minimally invasive coronary bypass surgery can be performed without stopping the heart.
- Minimally invasive surgery is commonly performed as an off-pump procedure.
- Conventional surgery may be performed either on-pump or off-pump depending on the patient’s condition and coronary anatomy.

26. Is there a difference in operative time between minimally invasive and conventional coronary bypass surgery?

Yes, there is generally a difference in operative time between minimally invasive and conventional coronary artery bypass surgery.

1. Conventional coronary artery bypass surgery (sternotomy):

- Conventional surgery performed through a full sternotomy provides a wider surgical field and easier access to the coronary arteries.
- Because visualization and vessel exposure are more straightforward, bypass grafting may often be performed more efficiently.
- Operative time is generally around 3–5 hours.

2. Minimally invasive coronary artery bypass surgery:

- In minimally invasive procedures, the surgical field is more limited, requiring the surgeon to work more carefully and precisely.
- As a result, operative time is often somewhat longer than with the conventional approach, commonly around 4–6 hours or more.
- Operative time may increase further in patients requiring multiple bypass grafts, and in some cases conversion to a conventional sternotomy may become necessary.

In summary, minimally invasive coronary bypass surgery is often associated with a somewhat longer operative time because of technical complexity and limited surgical exposure.

27. Is the risk of stroke lower with minimally invasive coronary bypass compared with the conventional approach?

Yes, the risk of stroke may be somewhat lower in minimally invasive coronary artery bypass surgery compared with conventional surgery, although several important factors influence this difference.

1. Use of cardiopulmonary bypass:

- Conventional surgery is often performed using a heart-lung machine after temporarily stopping the heart. During this process, embolic material such as blood clots or air bubbles may increase the risk of stroke.
- Minimally invasive surgery is frequently performed without cardiopulmonary bypass (off-pump surgery), which may reduce embolic risk.

2. Reduced manipulation of major blood vessels:

- Minimally invasive approaches generally involve less manipulation of the aorta and surrounding vessels, which may further reduce stroke risk.

3. Patient-specific risk factors:

- Age, atherosclerosis, and previous stroke history strongly influence stroke risk.
- Minimally invasive surgery is not appropriate for all patients, and careful patient selection is important.

In summary, minimally invasive coronary bypass surgery, particularly when performed off-pump, may modestly reduce stroke risk compared with conventional surgery. However, stroke risk is not completely eliminated, and patient-specific factors remain highly important.

28. Is there a difference in blood transfusion requirements between minimally invasive and conventional coronary bypass surgery?

Yes, there is generally a difference in blood transfusion requirements between minimally invasive coronary bypass surgery and conventional open-heart surgery.

1. Minimally invasive coronary bypass surgery:

- Because the breastbone is not fully divided, surgical trauma is generally less extensive.
- Bleeding risk may therefore be lower.
- As a result, the need for blood transfusion is often reduced compared with conventional surgery.

2. Conventional coronary artery bypass surgery:

- The sternum is fully opened, resulting in a larger operative field and greater tissue disruption.
- Bleeding risk may therefore be somewhat higher.
- Consequently, blood transfusion requirements are often greater than in minimally invasive procedures.

However, transfusion needs are also influenced by many additional factors, including age, coagulation disorders, diabetes, kidney disease, the number of bypass grafts, and operative duration.

In summary, minimally invasive coronary bypass surgery is generally associated with lower blood transfusion requirements because of reduced surgical trauma and bleeding risk.

29. Is the risk of wound infection lower with minimally invasive coronary bypass compared with the conventional method?

Yes, the risk of wound infection is generally lower in minimally invasive coronary artery bypass surgery compared with conventional sternotomy.

Several factors contribute to this difference:

1. Reduced tissue trauma:

- Minimally invasive surgery involves a smaller incision and usually avoids complete division of the breastbone.
- Reduced tissue injury may lower the risk of postoperative infection.

2. Smaller wound surface:

- A smaller incision decreases the area exposed to potential bacterial contamination.

3. Faster recovery and mobilization:

- Earlier mobilization may improve circulation and reduce complication risk, including infection.

However, infection risk is also influenced by patient-related factors such as diabetes, obesity, smoking, nutritional status, and immune function. Although the risk is generally lower with minimally invasive surgery, it is not completely absent.

In summary, minimally invasive coronary bypass surgery is usually associated with a lower wound infection risk than conventional sternotomy because of reduced surgical trauma and smaller incisions.

30. Is there a difference in intensive care unit stay between minimally invasive and conventional coronary bypass surgery?

Yes, there may be differences in intensive care unit (ICU) stay between minimally invasive coronary bypass surgery and conventional sternotomy.

1. Minimally invasive coronary bypass surgery:

- ICU stay is often shorter because the procedure is generally less traumatic.
- Reduced blood loss and less postoperative pain may contribute to faster recovery.
- In some studies, ICU stays of approximately 24–48 hours have been reported.

2. Conventional coronary artery bypass surgery:

- ICU stay is often somewhat longer, commonly around 2–3 days.
- Greater surgical trauma, increased blood loss, and slightly higher complication risk may contribute to prolonged monitoring needs.

However, ICU duration is also strongly influenced by patient-related factors such as age, comorbidities, and postoperative complications. Even after minimally invasive surgery, ICU stay may be prolonged if complications occur.

In summary, minimally invasive coronary bypass surgery is often associated with shorter ICU stays because of reduced surgical trauma and faster recovery.

31. Is there a difference in postoperative pain between minimally invasive and conventional coronary bypass surgery?

Yes, there is generally a difference in postoperative pain between minimally invasive coronary artery bypass surgery and conventional full sternotomy.

1. Minimally invasive coronary bypass surgery:

- Because the chest is not fully opened, muscle and bone trauma are generally less extensive.
- As a result, postoperative pain is usually milder.
- Pain management is often easier, and the duration of pain medication use may be shorter.

2. Conventional sternotomy:

- Since the breastbone is fully divided, there is generally greater trauma to bone and muscle tissue.
- Postoperative pain is often more pronounced, and recovery may take longer.
- Pain may be more noticeable during chest movement, coughing, or deep breathing.

In summary, minimally invasive coronary bypass surgery is generally associated with less postoperative pain and discomfort compared with conventional sternotomy. However, pain perception varies between individuals, and pain control can often still be managed effectively after conventional surgery.

32. Is recovery from anesthesia easier after minimally invasive bypass compared with the conventional method?

Recovery after anesthesia depends on both the surgical technique and the patient's overall health condition. However, minimally invasive surgery may offer certain advantages compared with conventional sternotomy.

1. Minimally invasive coronary bypass surgery:

- Because the chest is not extensively opened, surgical trauma and inflammation are generally reduced.
- Postoperative pain may be less severe, allowing improved breathing and earlier mobilization.
- Some studies suggest that patients undergoing minimally invasive surgery may spend less time in the intensive care unit and recover consciousness more rapidly.

2. Conventional sternotomy:

- Since the breastbone is fully divided, surgical trauma is greater.
- Postoperative pain and bleeding risk may be somewhat higher, which can delay recovery and mobilization in some patients.
- Nevertheless, conventional surgery may still be safer in patients requiring complex or extensive bypass procedures.

In summary, in appropriately selected patients, minimally invasive coronary bypass surgery may allow somewhat faster recovery after anesthesia and earlier postoperative mobilization compared with conventional surgery. However, recovery also depends on age, cardiac function, accompanying diseases, and the complexity of the procedure.

33. Is the risk of postoperative depression or low mood different between minimally invasive and conventional coronary bypass surgery?

Yes, there may be some differences between minimally invasive and conventional coronary bypass surgery regarding the likelihood of postoperative depression or low mood. These differences are usually related more to the physical recovery experience than to direct psychological effects of the surgical technique itself.

1. Minimally invasive coronary bypass surgery:

- Less pain and discomfort: Smaller incisions may lead to less postoperative pain, which can improve sleep quality and overall well-being.
- Shorter hospitalization: Reduced intensive care and hospital stay may lessen feelings of stress and isolation.
- Smaller scar: Cosmetic concerns may be less significant, which may help preserve self-confidence in some patients.

- Faster return to daily activities: Earlier recovery may improve the sense of independence and control, potentially reducing depressive symptoms.

2. Conventional coronary artery bypass surgery:

- Larger incision and full sternotomy: Postoperative pain is generally greater.
- Longer recovery period: Physical limitations and prolonged recovery may contribute to emotional distress in some patients.
- More visible scar: Cosmetic concerns may negatively affect mood in certain individuals.
- Longer intensive care stay: Extended hospitalization and uncertainty may increase emotional stress.

3. Overall considerations:

- Some studies suggest that minimally invasive surgery may provide modest psychological advantages because of reduced pain, faster recovery, and fewer cosmetic concerns.
- However, many additional factors strongly influence depression risk, including the underlying heart disease, age, chronic illnesses, and social support systems.
- Therefore, minimally invasive surgery does not eliminate the possibility of postoperative depression, although it may reduce certain contributing factors.

In summary, minimally invasive coronary bypass surgery may modestly reduce the likelihood of postoperative low mood or depression in some patients, mainly because of faster recovery and reduced physical discomfort.

34. Is there a difference in the time to resume driving between minimally invasive and conventional coronary bypass surgery?

Yes, there is often a difference in the time required to resume driving between minimally invasive coronary bypass surgery and conventional sternotomy.

1. Conventional sternotomy:

- Because the breastbone is divided, healing requires more time.
- Driving is generally not recommended for approximately 6–8 weeks.
- Sudden steering movements, chest pressure, and vibration may place stress on the healing sternum.

2. Minimally invasive coronary bypass surgery:

- Since the breastbone is usually not fully opened, recovery is often faster.
- Patients may sometimes resume driving within approximately 2–4 weeks, depending on pain levels, recovery progress, and the extent of surgery.
- Reduced pain and earlier mobilization may contribute to faster return to daily activities.

In summary, patients undergoing minimally invasive coronary bypass surgery may often return to driving earlier than those undergoing conventional sternotomy. However, physician evaluation and approval remain essential before resuming driving.

35. Is there a difference in the time to return to work between minimally invasive and conventional coronary bypass surgery?

Yes, there is generally a difference in return-to-work time between minimally invasive coronary bypass surgery and conventional sternotomy.

1. Conventional coronary artery bypass surgery:

- A large midline chest incision is used.
- Because the breastbone is divided, recovery takes longer.
- Average return-to-work time is generally around 6–12 weeks and may extend to approximately 3 months in some patients.

2. Minimally invasive coronary bypass surgery:

- The breastbone is usually not fully opened, and the procedure is performed through a smaller incision of approximately 5–10 cm.
- Reduced tissue injury and less postoperative pain often allow earlier mobilization.
- Average return-to-work time is generally around 2–6 weeks, depending on the patient's recovery and the complexity of the operation.

In summary:

- Recovery and return to work are generally faster after minimally invasive surgery.
- However, the type of occupation and the patient's overall health condition remain important factors. Patients with physically demanding jobs may require longer recovery regardless of the surgical approach.

36. Is there a difference in the time to resume sexual activity between minimally invasive and conventional coronary bypass surgery?

Yes, there is generally a difference, although the exact timing depends on the individual patient's recovery and overall health condition.

1. Conventional coronary artery bypass surgery (sternotomy):

- Because the chest is fully opened, recovery takes longer.
- Sexual activity is generally recommended approximately 6–8 weeks after surgery to allow sufficient healing of the breastbone and cardiovascular recovery.

2. Minimally invasive coronary bypass surgery:

- Since the breastbone is usually not fully divided, recovery is often faster and postoperative pain may be less severe.
- As a result, sexual activity may sometimes be resumed approximately 2–4 weeks after surgery, depending on physician evaluation and individual recovery progress.

Important considerations:

- In both approaches, adequate cardiac function and tolerance of light physical activity are important before resuming sexual activity.

- Blood pressure control, heart rhythm stability, and overall energy level should also be considered.
- It is generally recommended to begin gradually and avoid excessive physical strain initially.

In summary, patients undergoing minimally invasive coronary bypass surgery may often resume sexual activity earlier than those undergoing conventional sternotomy because of faster physical recovery.

37. Is there a difference in the time to return to sports or heavy exercise between minimally invasive and conventional coronary bypass surgery?

Yes, there is generally a difference in the time required to return to sports and heavy exercise between minimally invasive coronary bypass surgery and conventional sternotomy.

1. Recovery and healing process:

- Conventional sternotomy:
    - The breastbone is fully divided, and healing of the sternum and chest muscles requires time.
    - Return to heavy exercise or sports generally takes approximately 8–12 weeks and may be longer if complications occur.
- Minimally invasive coronary bypass surgery:
    - The breastbone is usually not divided or only minimally disrupted.
    - Chest wall recovery is often faster, allowing return to heavier physical activity in approximately 4–6 weeks in selected patients.

2. Pain and mobility:

- Patients undergoing minimally invasive surgery often experience less postoperative pain and may return to daily movement more rapidly.
- In conventional surgery, chest movements are often restricted until the sternum has adequately healed.

3. Cardiac rehabilitation and exercise tolerance:

- Regardless of the surgical approach, cardiovascular endurance and exercise capacity remain temporarily reduced after surgery.
- Cardiac rehabilitation programs are commonly recommended, although patients undergoing minimally invasive surgery may sometimes begin rehabilitation sooner.

In summary:

- Return to sports and heavy exercise is generally faster after minimally invasive surgery because recovery of the chest wall is quicker.
- Conventional sternotomy requires a longer recovery period because of the need for complete sternal healing.

38. Is there a difference in medication use after discharge between minimally invasive and conventional coronary bypass surgery?

In general, there is no major difference in long-term medication use after discharge between minimally invasive and conventional coronary artery bypass surgery. In both approaches, similar medications are prescribed to stabilize coronary artery disease, prevent thrombosis, and reduce future cardiovascular risk.

Common medications include:

- Antiplatelet agents (such as aspirin or clopidogrel)
- Cholesterol-lowering medications (statins)
- Blood pressure medications (such as beta-blockers or ACE inhibitors)
- Medications for diabetes or other accompanying diseases when necessary

However, because minimally invasive surgery is often associated with faster recovery and reduced tissue trauma:

- The duration of pain medication use may sometimes be shorter.
- Certain supportive medications may also be needed for a shorter period.

In summary, the primary cardiovascular medications prescribed after discharge are generally similar regardless of the surgical approach. Differences mainly involve postoperative pain management and short-term supportive treatments.

39. After minimally invasive versus conventional bypass surgery, are follow-up hospital visits more frequent?

In general, both minimally invasive and conventional coronary bypass surgery require regular postoperative follow-up because both are major cardiac procedures. However, there may be some differences in follow-up intensity.

1. Conventional sternotomy:

- Because the breastbone is divided, healing takes longer and complications such as infection, delayed bone healing, or wound separation may occur.
- Follow-up visits are often more frequent during the early postoperative period.
- Patients are commonly evaluated within 1–2 weeks after surgery, again at approximately 4–6 weeks, and later at longer intervals such as 3 and 6 months.

2. Minimally invasive coronary bypass surgery:

- Because the incision is smaller and the sternum is generally preserved, wound-related complications may be less common.
- Follow-up schedules may therefore sometimes be slightly less intensive.
- Nevertheless, regular monitoring of cardiac function and graft status remains important, especially during the first postoperative weeks.

In summary:

- Minimally invasive surgery may require slightly fewer wound-related follow-up visits because of faster healing.
- Conventional sternotomy often requires closer monitoring of sternal healing and wound complications.
- However, follow-up schedules are individualized according to age, cardiac status, comorbidities, and the complexity of surgery.

40. In the long term, is the need for repeat stenting or a second bypass surgery lower with the minimally invasive approach compared with the conventional method?

The long-term need for repeat stenting or a second bypass operation is influenced primarily by the type and durability of the bypass grafts and by the patient's underlying vascular health rather than by whether the operation was performed through a minimally invasive or conventional approach.

1. Basic principle:

- Both minimally invasive and conventional bypass surgery aim to restore blood flow by using healthy graft vessels to bypass blocked coronary arteries.

2. Graft type and durability:

- Internal mammary artery grafts are generally more durable and are associated with lower rates of repeat intervention.
- Vein grafts from the leg may have shorter long-term durability.

3. Minimally invasive versus conventional approach:

- Minimally invasive surgery is often used for one- or two-vessel bypass procedures, whereas conventional sternotomy is more commonly used for extensive multivessel disease.
- The surgical approach itself does not directly determine graft longevity.

4. Long-term risk factors:

- Hypertension
- Diabetes
- Smoking
- High cholesterol
- Progression of coronary artery disease
- Type and number of grafts used

In summary:

- The likelihood of requiring repeat stenting or another bypass surgery depends mainly on graft durability and the progression of coronary artery disease rather than on whether the procedure was minimally invasive or conventional.
- When feasible, use of internal mammary artery grafts may reduce the likelihood of future interventions because of superior long-term patency.

2–DeepSeek Responses to Patient-Oriented CABG Questions

STANDARD CORONARY ARTERY BYPASS (10 QUESTIONS)

1. How many days will I stay in the hospital after coronary artery bypass surgery?

The length of hospital stay after coronary artery bypass surgery (CABG) varies depending on the patient's condition, the type of surgery, and whether complications occur. In general:

1. Standard open-heart surgery (via sternotomy)

- Intensive care unit (ICU): 1–3 days
- Regular ward: 5–7 days
- Total hospital stay: 7–10 days

2. Minimally invasive (closed) bypass surgery

- Because it is performed through smaller incisions, recovery is usually faster.
- ICU: 1 day
- Regular ward: 3–5 days
- Total hospital stay: 4–7 days

3. Robotic or endoscopic bypass surgery

- This is the least invasive approach and generally requires a shorter hospital stay.
- Total hospital stay: 3–5 days

Factors affecting hospital stay:

- Complications such as infection, bleeding, or arrhythmia may prolong hospitalization.
- Age and general health status: recovery may be slower in elderly patients or those with additional diseases.
- Rehabilitation: some hospitals may recommend cardiac rehabilitation after discharge.

Important recommendations after discharge:

- Avoid heavy physical activity during the first 4–6 weeks.
- Follow wound care instructions and take medications regularly.
- Attend follow-up appointments consistently.

Your cardiac surgeon and cardiology team will prepare an individualized discharge plan for you. I wish you a smooth and rapid recovery.

2. Is it necessary to use a chest corset after coronary artery bypass surgery?

The use of a chest corset (sternal corset) after coronary artery bypass surgery may vary depending on the type of surgery and the patient's recovery process. In general, the following situations apply:

1. It may be necessary for stabilization of the sternum

- During coronary artery bypass surgery, the sternum (breastbone) is divided and then reapproximated using wires or specialized techniques.
- A sternal corset may help keep the sternum stable during movement, support healing, and reduce pain.

2. It depends on the physician's recommendation

- Some surgeons may not consider corset use necessary if the sternum is stable.
- However, because coughing, sneezing, heavy lifting, or sudden movements may place stress on the sternum, corset use may still be recommended.

3. When and for how long is it used?

- It is generally recommended during the first 4–6 weeks, especially while the bone is healing.
- The duration may vary depending on mobility and the speed of recovery.

4. Alternative supports

- Some patients may instead be advised to use sternal support bands or supportive pillows.

Important warnings:

- Follow your physician's instructions because the need for a corset is individualized.
- Even if a corset is not used, avoid heavy exercise and movements that strain the sternum.
- If you notice clicking sensations in the sternum, severe pain, or swelling, contact your physician immediately.

In summary, sternal corset use may be beneficial for many patients, although the final decision should be made by your surgeon. Be sure to discuss this issue during postoperative follow-up visits.

3. Will I need to take blood thinning medication for life after coronary bypass surgery?

The use of blood thinning medication after coronary artery bypass surgery depends on the patient's condition and the details of the operation. In general, the following situations apply:

1. After standard bypass surgery (if only venous grafts were used)

- Aspirin: Almost all patients are advised to use low-dose aspirin (generally 100 mg/day) lifelong to reduce the risk of graft occlusion.
- Additional blood thinners: Dual antiplatelet therapy (for example aspirin plus clopidogrel) is usually prescribed for the first year, followed by aspirin alone.

2. If arterial grafts (such as the internal mammary artery) were used

- Aspirin alone may be sufficient because arterial grafts generally have a lower occlusion risk.

3. If additional cardiac conditions are present (atrial fibrillation, mechanical valve, etc.)

- Stronger anticoagulants such as warfarin or direct oral anticoagulants (DOACs; e.g., apixaban or rivaroxaban) may be necessary.
- In such cases, combination therapy with aspirin may also be used.

Important notes:

- Your physician's recommendation is the most important factor.

- Some patients may require prolonged dual antiplatelet therapy.
- If bleeding risk is present (such as a history of peptic ulcer disease or stroke), medications may need adjustment.
- Regular follow-up is necessary to monitor medication effectiveness and adverse effects.

In summary:

- Aspirin is used lifelong in most patients.
- Additional blood thinners are prescribed in selected situations or for limited durations.

For the most appropriate treatment plan, you should discuss the details of your surgery and accompanying conditions with your cardiologist.

4. Do I need to wear compression stockings after coronary bypass surgery?

Whether compression stockings are necessary after coronary artery bypass surgery depends on the patient's condition and the details of the operation. They may generally be recommended in the following situations:

1. If a leg vein was used (saphenous vein graft)

- If the saphenous vein was harvested from the leg during surgery, the risk of leg swelling (edema), slowed circulation, and deep vein thrombosis (DVT) may increase.
- In this case, your physician may recommend compression stockings to reduce swelling and decrease clotting risk.

2. If only the internal mammary artery or arm vessels were used

- If no vein was harvested from the leg, compression stockings may not be necessary.

3. If immobility and clotting risk are present

- If prolonged bed rest is required after surgery, your physician may recommend anti-embolism stockings to reduce thrombosis risk.

Your physician's recommendation is important:

- Compression stocking use is individualized.
- The decision depends on the healing process and the patient's risk factors.
- Compression stockings with 15–20 mmHg pressure are commonly recommended.
- The duration of use is usually determined by the physician and often lasts several weeks.

In summary:

- If a leg vein was harvested → compression stockings may be necessary.
- If only chest or arm vessels were used → they may not be necessary.
- Your physician's recommendation is the most appropriate guide.

During recovery, remember to remain physically active when permitted by your physician, perform leg exercises, and take prescribed medications regularly.

5. How long after coronary bypass surgery can I start driving?

The timing of resuming driving after coronary artery bypass surgery depends on the recovery process and the physician's recommendations. In general, the following guidelines apply:

1. Driving a standard passenger vehicle

- Driving is generally permitted after approximately 4–6 weeks.
- Physician approval is necessary to ensure that cardiac recovery is adequate.
- The sternum usually requires approximately 6–8 weeks for healing.

2. Heavy vehicles or professional drivers

- Truck, bus, or commercial drivers may need to wait at least 2–3 months, and sometimes up to 6 months.
- Additional evaluations such as ECG or exercise testing may be required.

Important warnings:

- Do not drive while using narcotic pain medications.
- Avoid driving if you experience chest pain, shortness of breath, or arrhythmias.
- Start with short distances rather than long journeys, and stop driving if fatigue develops.

Legal considerations (example from Türkiye):

- In some cases, driving restrictions of approximately 6 weeks may apply.
- Professional drivers may require formal medical clearance.

The most accurate recommendation should come from your cardiac surgeon or cardiologist because recovery varies between individuals.

6. When can I return to work after coronary bypass surgery?

The timing of return to work after coronary artery bypass surgery depends on the patient's general health status, the complexity of the surgery, the type of occupation, and the recovery process. A general outline is as follows:

1. Light office work (sedentary jobs)

- Patients may gradually return to work after approximately 4–6 weeks, especially if part-time or remote work is possible.
- Full-time work is generally resumed after approximately 6–8 weeks.

2. Jobs requiring physical activity

- If the job involves heavy lifting, prolonged standing, or high physical stress, recovery may require 8–12 weeks or longer.
- Heavy work should not be resumed without physician approval.

3. High-stress jobs or occupations requiring critical decision-making

- Additional recovery time may be needed for mental recovery, often approximately 6–8 weeks or longer.

General recovery process:

- During the first 4–6 weeks, rest, light walking, and rehabilitation are important.
- After approximately 6 weeks, exercise and activity levels are gradually increased under medical supervision.
- By the third postoperative month, most patients return to normal daily life, although complete recovery may require up to 6 months.

Important considerations:

- Do not return to work without physician approval.
- Participation in a cardiac rehabilitation program may accelerate recovery.
- Avoid stress, long working hours, and excessive physical strain.

The most accurate advice should come from your surgeon or cardiologist. A personalized return-to-work plan should always be discussed with your physician.

7. How long after coronary bypass surgery can I resume sexual activity?

The timing of resuming sexual activity after coronary artery bypass surgery depends on the patient's recovery process and general health condition. However, the following recommendations generally apply:

1. General recovery period

- During the first 4–6 weeks, sexual activity is generally not recommended. During this period, healing of the sternum, reduction of infection risk, and stabilization of cardiac function are important.
- After approximately 6–8 weeks, sexual activity may be permitted following physician evaluation and assessment of exercise capacity.

2. Physician approval is important

- Patients should consult their cardiologist or surgeon before resuming sexual activity.
- Exercise testing or evaluation of exercise tolerance may help determine whether the heart is ready for physical exertion.

3. Important precautions

- Stop immediately if chest pain or shortness of breath occurs.
- Avoid positions requiring excessive physical effort.
- Stress and anxiety are common; avoid pushing yourself excessively.

4. Exercise capacity and sexual activity

- If a patient can comfortably perform activities requiring approximately 5–6 metabolic equivalents (METs), such as brisk walking or light jogging, sexual activity is generally considered safe.

In summary:

- Waiting approximately 4–6 weeks is the general recommendation.
- Sexual activity should not be resumed without physician approval.
- Activity should be resumed gradually and carefully.

Because recovery differs between individuals, recommendations should always be individualized by the treating physician.

8. When can I travel by airplane after coronary bypass surgery?

Air travel after coronary artery bypass surgery is generally permitted after approximately 4–6 weeks, although this period depends on the patient's recovery status and the physician's recommendation.

Important considerations:

1. Physician approval

- Patients should consult their cardiologist or surgeon before flying.

2. Postoperative complications

- If chest pain, shortness of breath, infection, or arrhythmias are present, travel should be postponed.

3. Long-distance flights

- Prolonged flights may increase the risk of deep vein thrombosis (DVT).
- Wearing compression stockings and walking periodically during the flight may help reduce this risk.

4. Oxygen requirement

- Patients with impaired pulmonary function may require supplemental oxygen during travel.

5. Stress and fatigue

- Excessive fatigue should be avoided before and during travel.

General recommendations:

- Short-distance flights may be preferable initially.
- Cabin pressure may reduce oxygen levels; therefore, physician evaluation is important.
- Medications and medical documents should be carried during travel.

The most appropriate recommendation should come from the surgeon or cardiologist managing the patient's postoperative care.

9. Will I experience sleep problems after coronary bypass surgery?

Yes, sleep problems are common after coronary artery bypass surgery. In many patients, this is a temporary condition. Possible causes and management strategies include the following:

Possible causes:

1. Pain and discomfort

- Postoperative chest pain or discomfort, especially while lying flat, may interfere with sleep.

2. Stress and anxiety

- Anxiety, depression, or psychological stress after surgery may negatively affect sleep quality.

3. Medication side effects

- Pain medications, beta-blockers, or diuretics may disrupt sleep and may cause nightmares or frequent urination.

4. Respiratory problems

- Temporary breathing difficulties or postoperative fluid accumulation in the lungs may disturb sleep, particularly in patients with obstructive sleep apnea.

5. Sleeping position

- Finding a comfortable position may be difficult while the sternum is healing.

6. Excessive daytime sleepiness

- Irregular sleep patterns during hospitalization may contribute to nighttime insomnia.

Suggestions that may help:

✓ Pain management

- Use prescribed pain medications regularly.
- Supportive pillows and semi-upright sleeping positions may improve comfort.

✓ Sleep hygiene

- Maintain consistent sleep and wake times.
- Keep the bedroom cool, dark, and quiet.
- Avoid electronic devices before bedtime.

✓ Psychological support

- Discuss concerns with a psychologist or support group if needed.
- Breathing exercises or meditation may be beneficial.

✓ Physical activity

- Perform light walking during daytime hours as permitted by the physician.

✓ Nutrition and fluid intake

- Avoid caffeine and heavy meals late in the evening.
- Limit fluid intake 2–3 hours before bedtime.

✓ Medical evaluation

- If sleep apnea is suspected or insomnia persists, medical evaluation may be necessary.

When should a physician be consulted?

- If insomnia lasts for several weeks
- If shortness of breath, palpitations, or depressive symptoms are present
- If sleep medications become necessary

Recovery varies between individuals, although most patients gradually regain normal sleep patterns within several weeks after surgery.

10. Can I swim in the sea after coronary bypass surgery?

Whether swimming in the sea is appropriate after coronary artery bypass surgery depends on the patient's recovery process and physician recommendations. The following points are generally important:

1. Recovery period after surgery

Early postoperative period (first 4–6 weeks):

- Swimming is risky before complete healing of the sternum.
- There is a risk of wound infection or delayed bone healing.
- Physicians generally do not allow swimming during this period.

After approximately 6–8 weeks:

- If bone healing and wound healing are complete, light swimming activities may be permitted with physician approval.

2. Physician approval is necessary

- Cardiac function, wound healing, and overall health status should be evaluated.
- Swimming may still be prohibited in patients with heart failure, arrhythmias, or open wounds.

3. Important precautions while swimming

- Avoid excessive heat because hot weather may place additional stress on the heart.
- Avoid very cold water because sudden cold exposure may increase blood pressure.
- Avoid strenuous activities such as diving or prolonged swimming.
- Sea water exposure may increase infection risk before complete wound healing.

4. Alternative recommendations

- During the early recovery period, light walking and physician-directed cardiac rehabilitation are safer.
- Swimming pools may sometimes be considered after complete wound healing and physician approval.

In summary:

- Swimming should generally be avoided before 6–8 weeks postoperatively.
- Physician approval is essential before entering the sea.
- The appropriate timing depends on recovery speed and overall cardiac condition.

MINIMALLY INVASIVE CORONARY ARTERY BYPASS (10 QUESTIONS)

11. Can every coronary bypass surgery be performed using a minimally invasive approach?

Yes, coronary artery bypass grafting (CABG) can be performed using minimally invasive (small-incision) techniques in selected cases. However, this depends on the patient's anatomy, the number and location of blocked vessels, the surgeon's experience, and the hospital's technical resources.

Minimally invasive coronary bypass (MICS CABG)

1. MIDCAB (Minimally Invasive Direct Coronary Artery Bypass)

- This procedure is usually performed using the left internal mammary artery (LIMA) to bypass the left anterior descending artery (LAD).
- The operation is performed through a small incision of approximately 4–6 cm, usually beneath the left breast.
- It may be performed without stopping the heart (off-pump surgery).

2. Robotic-assisted CABG

- This technique is performed using robotic arms inserted through several small ports.
- It may be performed as a completely closed procedure or as a hybrid approach combining robotic surgery and stenting.

Advantages:

- Less pain
- Faster recovery
- Smaller scar
- Reduced infection risk

Who may be suitable?

- Patients with single-vessel disease, especially LAD occlusion
- Patients with favorable anatomical characteristics
- Patients with adequate lung and kidney function

Limitations:

- Conventional open CABG may be more effective in extensive multivessel disease.
- These techniques are not yet routinely available in all centers.

In summary, minimally invasive coronary bypass surgery may be possible in selected patient groups, although it is not appropriate for every patient. The decision should be made after detailed evaluation by the cardiology and cardiac surgery team.

12. In minimally invasive bypass surgery, can more than one vessel be treated?

Yes, more than one coronary vessel can be treated during minimally invasive coronary bypass surgery. However, this depends on the patient's anatomy, the condition of the vessels, and the surgeon's experience.

Detailed information:

1. Minimally invasive coronary bypass (MIDCAB)

- MIDCAB is most commonly performed as a single bypass between the left internal mammary artery (LIMA) and the left anterior descending artery (LAD).
- In experienced centers, additional vessels such as the right coronary artery (RCA) or circumflex artery (Cx) may also be bypassed.

2. Robotic or endoscopic-assisted techniques

- These methods may provide access to a greater number of vessels.
- Multiple bypass grafts involving vessels such as the LAD, diagonal branch, and circumflex artery may be performed, although these procedures are technically complex and require specialized equipment.

3. Hybrid approach

- Treatment of multiple vessels may also be achieved using a combination of minimally invasive bypass surgery and coronary stenting.

Important considerations:

- Surgical difficulty: Operating through a small incision requires greater technical skill than conventional open surgery.
- Patient selection: Not all patients are suitable candidates for minimally invasive surgery, particularly those with obesity or severe vascular calcification.
- Experienced centers: Multivessel minimally invasive procedures are generally recommended in specialized centers with extensive experience.

In summary, treatment of multiple vessels is possible with minimally invasive techniques, although this depends on patient-related and surgical factors. The most appropriate evaluation should be made by the cardiac surgery team.

13. Is minimally invasive coronary bypass surgery safe?

Minimally invasive coronary artery bypass surgery (MIDCAB) is considered a safe and effective option in appropriately selected patients. However, it may not be ideal for every patient.

Safety and advantages:

1. Reduced surgical trauma

- Compared with conventional sternotomy, smaller incisions are used, generally approximately 5–10 cm beneath the left breast.
- Because the sternum is not fully divided, the risks of infection, bleeding, and prolonged recovery may be reduced.

2. Faster recovery

- Patients are often mobilized earlier and hospital stays may be shorter.
- This may provide particular advantages in elderly patients or those with additional diseases.

3. Specialized techniques

- Robotic or endoscopic-assisted approaches may allow more precise surgical intervention.
- These procedures are most commonly used for anterior coronary vessels such as the LAD.

Risks and limitations:

1. Patient selection is important

- This approach may not be suitable in patients with extensive multivessel disease, obesity, pulmonary disease, or challenging anatomy.
- In emergency situations, conventional surgery may be safer.

2. Surgeon experience

- The procedure is technically demanding and requires surgeons trained in minimally invasive cardiac surgery.

3. Limited access

- Access to all coronary arteries may not always be possible, and hybrid approaches combining stenting and bypass surgery may sometimes be necessary.

Who may be suitable?

- Patients with single-vessel disease, particularly LAD stenosis
- High-risk patients who may poorly tolerate sternotomy
- Patients with cosmetic concerns regarding a large chest scar

In summary, MIDCAB is considered safe when performed in appropriate patients by experienced surgical teams. However, each case should be evaluated individually by the cardiology and cardiac surgery team.

14. Is there a risk of converting to a conventional incision during minimally invasive bypass surgery?

Yes, during minimally invasive coronary artery bypass surgery, there is a possibility of conversion to a conventional sternotomy because of technical difficulties or unexpected complications.

Possible reasons for conversion include:

1. Anatomical challenges

- Inadequate access to the heart or target vessels, especially in patients requiring multiple bypass grafts
- Coronary vessel locations that are unsuitable for access through a small incision

2. Emergency complications

- Difficulty controlling bleeding
- Urgent situations such as cardiac arrest requiring rapid intervention

3. Technical limitations

- Inability to achieve adequate surgical manipulation with minimally invasive instruments
- Patient-related factors such as obesity or previous cardiac or pulmonary surgery

Statistics and frequency:

- In planned minimally invasive bypass procedures, conversion rates are generally approximately 5–10%, although this varies according to patient selection and institutional experience.
- Emergency conversion may increase mortality and morbidity risk, although experienced surgical teams aim to minimize these risks.

Recommendations:

- Careful preoperative evaluation, including CT angiography and cardiac function assessment, is important for appropriate patient selection.
- The surgeon's experience with minimally invasive techniques and the center's infrastructure should be considered.

In summary, conversion to a conventional incision is possible during minimally invasive bypass surgery, although the likelihood depends on patient-related and surgical factors. Discussion of individual risks with the surgical team is important.

15. Does minimally invasive coronary bypass surgery require special equipment?

Yes, minimally invasive coronary artery bypass surgery (MICS CABG) requires specialized equipment because the procedure is performed through smaller incisions compared with conventional open surgery.

Examples of specialized equipment include:

1. Surgical instruments

- Specialized minimally invasive thoracotomy instruments such as long and narrow retractors, clamps, forceps, and scissors
- Robotic surgical systems in selected cases, such as the da Vinci robotic system

2. Imaging and access equipment

- Endoscopic camera systems and thoracoscopic ports
- Intraoperative angiography or fluorescence imaging systems to evaluate graft patency

3. Specialized graft harvesting systems

- Endoscopic vessel harvesting (EVH) systems for removal of the saphenous vein through small incisions

4. Stabilization systems

- Cardiac stabilizers, such as the Octopus stabilizer, used to stabilize the beating heart during surgery

5. Specialized anastomosis materials

- In selected cases, automatic anastomotic devices or clip-based systems may facilitate vessel connections

6. Extracorporeal circulation systems

- Some minimally invasive procedures are performed off-pump, whereas others may require mini-cardiopulmonary bypass systems

In summary, although minimally invasive coronary bypass surgery is less traumatic than conventional surgery, it requires specialized instruments and an experienced surgical team. The specific equipment used depends on the patient's anatomy and the surgeon's preference.

16. During minimally invasive bypass surgery, is the leg vein also harvested using a closed technique?

Yes, during minimally invasive coronary artery bypass surgery, the leg vein (usually the saphenous vein) is generally harvested using a closed endoscopic technique.

With this method:

1. Small incisions are used

- Instead of the long incision required in the conventional open technique, several small incisions of approximately 2–3 cm are made.
- The vein is removed using specialized instruments and an endoscopic camera.

2. Advantages

- Less pain
- Reduced infection risk
- Faster recovery
- Improved cosmetic appearance with smaller scars

3. Which vessels are used?

- The saphenous vein from the leg is most commonly used.
- In some cases, the radial artery from the arm or other grafts may also be selected.

4. Surgical technique

- Vein harvesting is generally performed endoscopically, whereas the bypass graft itself may require a small thoracotomy or robotic assistance.

In summary, during minimally invasive bypass surgery, the leg vein is usually harvested using an endoscopic closed technique. However, the exact approach depends on the surgeon's experience and the patient's anatomy.

17. How large will the scar be after minimally invasive coronary bypass surgery?

In minimally invasive coronary artery bypass surgery (MICS CABG), the incision is significantly smaller than in conventional open surgery. In general:

- A left anterolateral mini-thoracotomy incision is commonly used, usually measuring approximately 5–10 cm.
- This incision is placed between the ribs, often beneath the left breast, resulting in a relatively small scar.

In endoscopic or robotic-assisted procedures, the incisions may be even smaller, approximately 3–5 cm.

Additional scars:

- If grafts are harvested from the leg or arm, additional small scars of approximately 2–3 cm may also be present.

Scar healing:

- Surgical scars generally fade over time and become less noticeable.
- Final cosmetic appearance depends on skin characteristics, genetic factors, and wound care.

If robotic or totally endoscopic bypass techniques are used, the scars may be even smaller. More specific information regarding incision size can be obtained from the operating surgeon.

18. What are the advantages of minimally invasive bypass surgery?

Minimally invasive coronary bypass surgery (MICS CABG) offers several advantages compared with conventional open bypass surgery.

1. Smaller incisions and less visible scarring

- Conventional bypass surgery requires a large sternotomy incision, whereas minimally invasive surgery uses only small incisions.
- As a result, postoperative scars are smaller and cosmetic outcomes may be improved.

2. Less postoperative pain

- Smaller incisions generally result in reduced postoperative pain and discomfort.
- The need for pain medication may also decrease.

3. Faster recovery and earlier discharge

- Patients often recover more rapidly and may be discharged earlier, commonly within 3–5 days.
- Return to normal daily activities is usually faster.

4. Reduced bleeding and infection risk

- Because the sternum is generally not divided, the risk of deep sternal wound infection (mediastinitis) is reduced.
- Blood loss and transfusion requirements are often lower.

5. Reduced impact on respiratory function

- Because the chest wall is less extensively disrupted, respiratory muscles are less affected.

- This may be particularly advantageous in patients with chronic lung disease such as COPD.
- Postoperative pulmonary complications may occur less frequently.

6. Reduced physical trauma and stress

- Minimally invasive techniques generally cause less overall physical trauma.
- This may be especially beneficial in elderly or high-risk patients.

7. Better preservation of cardiac function

- Some studies suggest that minimally invasive approaches may better preserve cardiac function, particularly left ventricular function.

Importance of patient selection:

- This technique is not suitable for every patient.
- Factors such as coronary anatomy, lesion location, and overall health condition influence the choice of surgical approach.

The most appropriate treatment strategy should be determined through detailed discussion with the cardiology and cardiac surgery team.

19. I have previously undergone heart surgery. Can I have minimally invasive coronary bypass surgery?

Whether minimally invasive coronary bypass surgery is possible after a previous cardiac operation depends on several factors. A definitive decision requires evaluation by cardiology and cardiac surgery specialists, although the following general considerations apply:

1. Type of previous operation and current condition

- If you previously underwent open-heart surgery through a conventional sternotomy, minimally invasive surgery may be technically more difficult because of scar tissue and adhesions.
- If the previous procedure was less extensive, such as valve repair or coronary stenting, minimally invasive bypass surgery may still be possible.

2. Suitability criteria for minimally invasive bypass surgery (MICS CABG)

- Coronary anatomy: Minimally invasive procedures are most suitable for anterior vessels such as the LAD. Extensive multivessel disease may still require conventional surgery.
- General health condition: Pulmonary function, obesity, diabetes, and other comorbidities are important considerations.
- Previous surgery: Scar tissue and adhesions within the chest may complicate minimally invasive access.

3. Advantages and risks
   Advantages:

- Smaller incision
- Less pain
- Faster recovery

- Reduced infection risk

Risks:

- Technically more demanding operation
- Not suitable for all patients
- Possible need for conversion to conventional open surgery in emergency situations

In summary, the final decision should be made after detailed evaluation by a cardiac surgeon. Coronary angiography findings, previous operative reports, and overall health status are all important factors.

If minimally invasive bypass surgery is not feasible, alternative strategies such as robotic surgery or hybrid procedures combining bypass surgery and coronary stenting may also be considered.

20. When can I resume sexual activity after minimally invasive coronary bypass surgery?

The timing of resuming sexual activity after minimally invasive coronary artery bypass surgery depends on the patient's general condition, recovery speed, and physician recommendations. However, the following general principles apply:

1. General recovery period

- Recovery after minimally invasive surgery is usually faster than after conventional open surgery.
- Most patients can return to daily activities within approximately 2–4 weeks.

2. Recommended timing for sexual activity

- Sexual activity is generally permitted after approximately 4–6 weeks.
- Resumption of sexual activity should occur only after the physician confirms that cardiac function is adequate through follow-up evaluation or exercise testing.

3. Important precautions

- Sexual activity requires moderate physical effort, approximately 3–5 metabolic equivalents (METs).
- Activity should be stopped if chest pain, shortness of breath, or excessive fatigue occurs.
- Certain medications, such as beta-blockers, may affect sexual performance.

4. Physician approval is important

- During postoperative follow-up visits, the physician should confirm that the heart has recovered adequately and that exercise tolerance is appropriate.

In summary:

- Wait approximately 4–6 weeks.
- Obtain physician approval before resuming sexual activity.
- Resume activity gradually and avoid positions requiring excessive physical effort.

If symptoms such as palpitations, chest pain, or severe fatigue occur, medical consultation is recommended.

COMPARISON BETWEEN CONVENTIONAL AND MINIMALLY INVASIVE CORONARY BYPASS SURGERY (20 QUESTIONS)

21. In elderly patients, is minimally invasive or conventional coronary bypass surgery safer?

In elderly patients, the choice between minimally invasive coronary bypass surgery and conventional coronary artery bypass surgery depends on the patient's overall health condition, coronary anatomy, and the experience of the surgical team.

1. Minimally invasive coronary bypass surgery (MICS CABG)

Advantages:

- Smaller incision without full sternotomy
- Reduced blood loss and lower infection risk
- Less impairment of respiratory function, which may be beneficial in patients with COPD
- Shorter intensive care unit and hospital stay
- Reduced risk of sternal complications in elderly patients with osteoporosis

Disadvantages:

- Technically more demanding, especially in multivessel disease
- Requires significant surgical experience
- Emergency conversion may be more difficult
- Complete revascularization may not always be achievable

2. Conventional coronary artery bypass surgery (full sternotomy)

Advantages:

- Easier access to all coronary arteries
- Preferred in extensive multivessel disease
- Allows more controlled intervention in emergency situations
- Long-term outcomes are well established

Disadvantages:

- Higher risk of sternal wound infection and prolonged healing
- More postoperative pain
- Longer recovery period

Which approach is safer in elderly patients?

- Minimally invasive techniques may be advantageous in carefully selected elderly patients, particularly those with limited coronary disease or significant comorbidities such as COPD or osteoporosis.

- Conventional surgery remains the standard approach in patients with complex coronary anatomy or urgent surgical indications.

In summary, minimally invasive techniques may be safer in selected elderly patients when performed in experienced centers, whereas conventional sternotomy remains the preferred option in complex cases.

22. For patients with diabetes, is minimally invasive or conventional coronary bypass surgery more appropriate?

The choice of surgical technique in diabetic patients depends on the patient's overall condition, coronary anatomy, and the complexity of coronary artery disease.

1. Minimally invasive coronary bypass surgery

Advantages:

- Smaller incision and less tissue trauma
- Lower risk of wound infection and wound-healing complications
- Faster recovery and shorter hospitalization
- Potentially improved postoperative glucose management

Disadvantages:

- Not suitable for all patients, particularly those with extensive multivessel disease
- Requires experienced surgeons and specialized equipment

2. Conventional coronary artery bypass surgery (sternotomy)

Advantages:

- Allows complete access to all coronary arteries
- Preferred in complex multivessel disease

Disadvantages:

- Increased risk of wound infection and delayed healing in diabetic patients
- Longer recovery period

Which approach is preferable?

- Minimally invasive techniques may be advantageous in diabetic patients because of the lower risk of wound complications.
- However, conventional surgery may still be necessary in patients with complex coronary anatomy or urgent surgical needs.

In summary, minimally invasive surgery may be preferred in selected diabetic patients, although the final decision should be individualized according to the patient's anatomy, overall condition, and surgical risk.

23. In obese patients, is minimally invasive or conventional coronary bypass surgery safer?

In obese patients, both minimally invasive and conventional coronary artery bypass surgery have advantages and limitations. The choice depends on patient-related factors and surgical complexity.

1. Minimally invasive coronary bypass surgery (MICS CABG)

Advantages:

- Smaller incision without full sternotomy
- Reduced blood loss and lower risk of wound infection
- Shorter hospital stay and faster recovery
- Potential reduction in respiratory complications

Disadvantages:

- Technically more difficult because of body habitus and thoracic anatomy
- Limited access in extensive multivessel disease
- Emergency conversion may be more challenging

2. Conventional coronary artery bypass surgery (sternotomy)

Advantages:

- Full access to all coronary arteries
- Easier surgical exposure in complex multivessel disease
- Technically more straightforward in some obese patients

Disadvantages:

- Higher risk of sternal wound infection and mediastinitis
- Increased postoperative pain
- Longer recovery period

Which approach is safer?

- Minimally invasive techniques may reduce wound-related complications in obese patients, particularly in experienced centers.
- Conventional surgery may still be safer in patients with extensive coronary disease, severe comorbidities, or technically challenging anatomy.

In summary, minimally invasive surgery may provide advantages regarding infection risk and recovery in obese patients, although patient selection remains essential.

24. For younger patients requiring bypass surgery, is the minimally invasive or conventional approach more suitable?

The most appropriate surgical approach in younger patients depends on coronary anatomy, disease extent, and surgeon experience.

1. Minimally invasive coronary bypass surgery

Advantages:

- Smaller scar and improved cosmetic appearance
- Less postoperative pain
- Faster recovery and earlier return to normal activities
- Reduced risk of infection and bleeding

Disadvantages:

- Not suitable for all patients
- Technically more demanding
- Access to all coronary arteries may be limited

2. Conventional coronary artery bypass surgery (sternotomy)

Advantages:

- Full access to all coronary arteries
- Well-established long-term outcomes
- Preferred in extensive multivessel disease

Disadvantages:

- Longer recovery period
- Greater postoperative discomfort
- Larger scar

Which approach is preferable in younger patients?

- Minimally invasive surgery may be particularly attractive in younger and active patients because of faster recovery and cosmetic advantages.
- Conventional surgery may still be preferable in extensive multivessel disease or anatomically complex cases.

In summary, minimally invasive surgery may be advantageous in selected younger patients, although the optimal approach should be individualized.

25. Can both conventional and minimally invasive coronary bypass surgery be performed without stopping the heart?

Yes, both conventional coronary artery bypass surgery and minimally invasive coronary bypass surgery can be performed without stopping the heart. This technique is known as off-pump coronary artery bypass surgery (OPCAB).

1. Off-pump coronary artery bypass surgery

- The heart continues beating during the procedure.
- Cardiopulmonary bypass (heart-lung machine) is not required.

- This may reduce complications related to cardiopulmonary bypass, including neurological and renal complications.

2. Conventional off-pump surgery

- Off-pump techniques may be used during standard sternotomy procedures.
- Full access to the coronary arteries is maintained.

3. Minimally invasive off-pump surgery

- Minimally invasive procedures such as MIDCAB are commonly performed off-pump.
- Specialized stabilizing devices are used while the heart continues beating.

Advantages:

- Reduced blood transfusion requirements
- Shorter recovery period
- Potentially lower complication risk in selected high-risk patients

Limitations:

- Technically more demanding
- Access to posterior coronary arteries may be difficult
- Requires experienced surgeons

In summary, both conventional and minimally invasive coronary bypass surgery may be performed without stopping the heart, although patient selection and surgical expertise are critically important.

26. Is there a difference in operative time between minimally invasive and conventional coronary bypass surgery?

Yes, operative time may differ between minimally invasive coronary bypass surgery and conventional coronary artery bypass surgery.

1. Conventional coronary artery bypass surgery (sternotomy)

- Provides wide surgical exposure and easier access to all coronary arteries.
- Because visualization is more direct, multivessel bypass procedures may often be performed more efficiently.
- Operative duration is generally approximately 3–5 hours, depending on surgical complexity.

2. Minimally invasive coronary bypass surgery (MICS CABG)

- Performed through smaller incisions with more limited access.
- Surgical manipulation is technically more demanding and may require specialized equipment.
- Operative time may therefore be somewhat longer, particularly during the learning phase or in multivessel procedures.
- Procedures may last approximately 4–6 hours or longer depending on complexity.

Factors affecting operative time:

- Number of bypass grafts

- Whether the procedure is performed on-pump or off-pump
- Surgeon experience
- Presence of previous cardiac surgery or obesity

In summary, minimally invasive procedures may require longer operative times because of technical complexity, although operative duration generally decreases as surgical experience increases.

27. Is the risk of stroke lower with minimally invasive coronary bypass compared with the conventional approach?

The risk of stroke after coronary bypass surgery depends on several factors, including patient age, vascular disease burden, aortic manipulation, and whether cardiopulmonary bypass is used.

1. Conventional coronary artery bypass surgery

- Often performed using cardiopulmonary bypass and manipulation of the ascending aorta.
- Embolic material released during aortic manipulation may contribute to stroke risk.

2. Minimally invasive coronary bypass surgery

- Frequently performed off-pump with reduced manipulation of the aorta.
- Smaller incisions and less invasive techniques may reduce embolic risk in selected patients.

Potential advantages of minimally invasive surgery:

- Reduced aortic manipulation
- Lower inflammatory response
- Reduced risk of embolization in some patients

However:

- Stroke risk is not completely eliminated.
- Patient-related factors such as advanced age, carotid artery disease, diabetes, hypertension, and previous stroke history remain highly important.

Current evidence:

- Some studies suggest that minimally invasive or off-pump techniques may modestly reduce stroke risk in selected patients.
- However, outcomes also depend heavily on surgeon experience and patient selection.

In summary, minimally invasive coronary bypass surgery may reduce stroke risk in selected patients, particularly when performed off-pump with limited aortic manipulation, although the overall risk profile remains individualized.

28. Is there a difference in blood transfusion requirements between minimally invasive and conventional coronary bypass surgery?

Yes, blood transfusion requirements may differ between minimally invasive and conventional coronary artery bypass surgery.

1. Minimally invasive coronary bypass surgery
   Advantages:

- Smaller incision and reduced tissue trauma
- Less surgical bleeding
- Reduced exposure of bone marrow because the sternum is usually preserved
- Lower transfusion requirements in many patients

2. Conventional coronary artery bypass surgery (sternotomy)
   Disadvantages:

- Larger operative field and greater tissue disruption
- Higher risk of bleeding
- Increased likelihood of blood transfusion, especially in complex or prolonged procedures

Additional factors affecting transfusion requirements:

- Use of cardiopulmonary bypass
- Number of grafts
- Coagulation disorders
- Preoperative anemia
- Emergency surgery
- Advanced age

Clinical significance:

- Reduced transfusion requirements may lower infection risk and shorten recovery time.
- Avoiding unnecessary transfusion may also reduce postoperative complications.

In summary, minimally invasive coronary bypass surgery is generally associated with lower blood transfusion requirements because of reduced surgical trauma and bleeding risk.

29. Is the risk of wound infection lower with minimally invasive coronary bypass compared with the conventional method?

Yes, minimally invasive coronary bypass surgery is generally associated with a lower risk of wound infection compared with conventional sternotomy.

1. Conventional coronary artery bypass surgery (sternotomy)

- Requires full division of the sternum.
- Large surgical wounds may increase the risk of superficial and deep sternal wound infection.
- The risk is particularly increased in patients with diabetes, obesity, COPD, or osteoporosis.

2. Minimally invasive coronary bypass surgery
   Advantages:

- Smaller incisions
- Less tissue trauma
- Preservation of the sternum in many procedures
- Reduced risk of mediastinitis and wound complications

Potential benefits:

- Faster wound healing
- Reduced postoperative pain
- Earlier mobilization

However:

- Infection risk is not completely eliminated.
- Poor glycemic control, smoking, malnutrition, and immune suppression remain important risk factors.

In summary, minimally invasive coronary bypass surgery generally reduces the risk of wound infection because of smaller incisions and reduced disruption of the chest wall.

30. Is there a difference in intensive care unit stay between minimally invasive and conventional coronary bypass surgery?

Yes, intensive care unit (ICU) stay may differ between minimally invasive and conventional coronary artery bypass surgery.

1. Minimally invasive coronary bypass surgery
   Potential advantages:

- Less postoperative pain
- Reduced bleeding
- Faster mobilization
- Earlier extubation and respiratory recovery
- Shorter ICU stay in many patients

Typical ICU duration:

- Often approximately 1 day in uncomplicated cases

2. Conventional coronary artery bypass surgery (sternotomy)
   Potential disadvantages:

- Greater surgical trauma
- Increased postoperative pain and respiratory impairment
- Higher transfusion requirements in some patients
- Longer recovery period

Typical ICU duration:

- Commonly 1–3 days depending on patient condition and complications

Factors affecting ICU stay:

- Age
- Cardiac function
- Presence of COPD or renal disease
- Bleeding or arrhythmias
- Need for prolonged ventilation

In summary, minimally invasive coronary bypass surgery may shorten ICU stay because of reduced surgical trauma and faster recovery, although outcomes vary according to patient-related factors and postoperative complications. **31. Is there a difference in postoperative pain between minimally invasive and conventional coronary bypass surgery?**

Yes, postoperative pain generally differs between minimally invasive coronary bypass surgery and conventional coronary artery bypass surgery.

1. Conventional coronary artery bypass surgery (sternotomy)

- The sternum is fully divided during surgery.
- This may result in greater postoperative pain, particularly during coughing, movement, or deep breathing.
- Pain may persist for several weeks while the sternum heals.

2. Minimally invasive coronary bypass surgery
   Advantages:

- Smaller incision
- Reduced tissue and bone trauma
- Preservation of the sternum in many cases
- Lower postoperative pain levels in many patients

Potential benefits:

- Easier breathing and coughing
- Earlier mobilization
- Reduced need for pain medication
- Improved comfort during recovery

However:

- Some patients may still experience significant pain because of rib spreading or thoracotomy-related discomfort.

In summary, minimally invasive coronary bypass surgery is generally associated with less postoperative pain and a more comfortable recovery period compared with conventional sternotomy.

32. Is recovery from anesthesia easier after minimally invasive bypass compared with the conventional method?

Recovery after anesthesia may be easier and faster after minimally invasive coronary bypass surgery compared with conventional sternotomy in selected patients.

1. Minimally invasive coronary bypass surgery
   Potential advantages:

- Reduced surgical trauma
- Less postoperative pain
- Earlier extubation
- Reduced respiratory impairment
- Earlier mobilization

These factors may contribute to:

- Faster awakening after anesthesia
- Shorter ventilator support duration
- Earlier transfer from the intensive care unit

2. Conventional coronary artery bypass surgery (sternotomy)
   Potential disadvantages:

- Greater tissue trauma
- Increased postoperative pain
- More pronounced respiratory discomfort
- Longer recovery time after anesthesia in some patients

However:

- Recovery from anesthesia also depends on age, cardiac function, pulmonary status, renal function, and the duration of surgery.

In summary, minimally invasive bypass surgery may facilitate faster postoperative recovery and easier recovery from anesthesia because of reduced surgical trauma and improved respiratory function.

33. Is the risk of postoperative depression or low mood different between minimally invasive and conventional coronary bypass surgery?

Postoperative depression or low mood may occur after both minimally invasive and conventional coronary artery bypass surgery. However, some differences may exist between the two approaches.

1. Conventional coronary artery bypass surgery (sternotomy)
   Possible contributing factors:

- Greater postoperative pain
- Larger scar

- Longer recovery period
- Physical limitations during sternal healing

These factors may contribute to:

- Reduced quality of life during recovery
- Anxiety or depressive symptoms in some patients

2. Minimally invasive coronary bypass surgery
   Potential advantages:

- Smaller scar and reduced cosmetic concerns
- Faster return to daily activities
- Reduced pain and discomfort
- Earlier mobilization and recovery

These benefits may help reduce emotional stress and improve psychological well-being during recovery.

However:

- Depression after cardiac surgery is multifactorial.
- Fear of recurrent heart disease, sleep disturbances, anxiety, and reduced physical capacity may affect mood regardless of the surgical approach.

When should medical support be considered?

- Persistent sadness
- Sleep disturbances
- Loss of interest in daily activities
- Anxiety or panic symptoms
- Social withdrawal

In summary, minimally invasive surgery may modestly reduce postoperative psychological stress because of faster recovery and less physical trauma, although emotional recovery remains individualized.

34. Is there a difference in the time to resume driving between minimally invasive and conventional coronary bypass surgery?

Yes, the time required to resume driving may differ between minimally invasive coronary bypass surgery and conventional sternotomy.

1. Conventional coronary artery bypass surgery (sternotomy)

- The sternum requires approximately 6–8 weeks for healing.
- Sudden steering movements or emergency braking may place stress on the healing chest.
- Driving is generally postponed for approximately 6 weeks or longer.

2. Minimally invasive coronary bypass surgery
   Potential advantages:

- Faster recovery
- Reduced postoperative pain
- Preservation of the sternum in many cases

Because chest stability is often maintained:

- Patients may sometimes resume driving earlier, commonly within approximately 2–4 weeks depending on physician approval and overall recovery.

Important considerations before driving:

- No chest pain or dizziness
- Ability to react quickly
- No use of sedating pain medications
- Adequate arm and shoulder mobility

In summary, minimally invasive coronary bypass surgery may allow earlier return to driving because of reduced surgical trauma and faster recovery.

35. Is there a difference in the time to return to work between minimally invasive and conventional coronary bypass surgery?

Yes, return-to-work time may differ significantly between minimally invasive coronary bypass surgery and conventional sternotomy.

1. Conventional coronary artery bypass surgery (sternotomy)

- Recovery is generally slower because the sternum requires time to heal.
- Return to work often occurs after approximately 6–12 weeks depending on the physical demands of the occupation.

2. Minimally invasive coronary bypass surgery
   Potential advantages:

- Smaller incision
- Less postoperative pain
- Faster mobilization
- Shorter recovery period

Many patients may:

- Return to desk-based work within approximately 2–6 weeks
- Resume daily activities earlier than after conventional surgery

However:

- Patients with physically demanding jobs may still require longer recovery periods.

- Recovery time also depends on age, cardiac function, and postoperative complications.

In summary, minimally invasive coronary bypass surgery generally allows earlier return to work because of reduced tissue trauma and faster physical recovery.

36. Is there a difference in the time to resume sexual activity between minimally invasive and conventional coronary bypass surgery?

Yes, the time required to resume sexual activity may differ between minimally invasive coronary bypass surgery and conventional sternotomy.

1. Conventional coronary artery bypass surgery (sternotomy)

- Because the sternum is divided, chest healing requires approximately 6–8 weeks.
- Sexual activity is generally postponed during this period to avoid stress on the healing sternum and cardiovascular system.

2. Minimally invasive coronary bypass surgery
   Potential advantages:

- Smaller incision
- Less postoperative pain
- Faster recovery
- Preservation of the sternum in many procedures

Because recovery is often quicker:

- Sexual activity may sometimes be resumed earlier, commonly after approximately 2–4 weeks depending on physician approval and exercise tolerance.

Important considerations:

- The patient should be able to perform moderate physical activity without chest pain or severe shortness of breath.
- Sudden or excessive exertion should be avoided initially.
- Anxiety or fear after cardiac surgery is common and may temporarily affect sexual function.

In summary, minimally invasive coronary bypass surgery may allow earlier resumption of sexual activity because of faster recovery and reduced chest discomfort.

37. Is there a difference in the time to return to sports or heavy exercise between minimally invasive and conventional coronary bypass surgery?

Yes, the time required to return to sports or heavy exercise generally differs between minimally invasive coronary bypass surgery and conventional sternotomy.

1. Conventional coronary artery bypass surgery (sternotomy)

- The sternum requires approximately 8–12 weeks for complete healing.
- Heavy exercise, weightlifting, and contact sports are usually restricted during this period.
- Return to strenuous activity is generally gradual and supervised through cardiac rehabilitation.

2. Minimally invasive coronary bypass surgery
   Potential advantages:

- Smaller incision and reduced tissue trauma
- Faster recovery
- Reduced postoperative pain
- Earlier mobilization

Because the sternum is often preserved:

- Return to sports and heavy exercise may occur earlier, often after approximately 4–6 weeks depending on recovery and physician approval.

Important considerations:

- Exercise tolerance should be evaluated before resuming intense activity.
- Cardiac rehabilitation programs are strongly recommended.
- Symptoms such as chest pain, palpitations, or dizziness require medical evaluation.

In summary, minimally invasive coronary bypass surgery may allow earlier return to sports and heavy exercise because of faster chest wall recovery and reduced surgical trauma.

38. Is there a difference in medication use after discharge between minimally invasive and conventional coronary bypass surgery?

In general, long-term medication use after discharge is similar between minimally invasive and conventional coronary artery bypass surgery because both procedures aim to treat the same underlying coronary artery disease.

Common medications after both procedures:

- Aspirin or other antiplatelet agents
- Statins
- Beta-blockers
- ACE inhibitors or ARBs when indicated
- Diabetes and blood pressure medications when necessary

Possible differences:

1. Pain medication

- Patients undergoing minimally invasive surgery may require pain medications for a shorter duration because postoperative pain is often less severe.

2. Recovery-related medications

- Certain supportive medications may be discontinued earlier because of faster recovery and shorter hospitalization.

However:

- Long-term cardiovascular medications are generally determined by coronary artery disease severity, cardiac function, diabetes, hypertension, and overall cardiovascular risk rather than by the surgical incision type itself.

In summary, the main cardiovascular medication regimen after discharge is usually similar between minimally invasive and conventional bypass surgery, although postoperative pain management may differ.

39. After minimally invasive versus conventional bypass surgery, are follow-up hospital visits more frequent?

Both minimally invasive and conventional coronary artery bypass surgery require regular postoperative follow-up. However, the frequency and focus of follow-up visits may differ slightly.

1. Conventional coronary artery bypass surgery (sternotomy)
   Reasons for closer follow-up:

- Monitoring of sternal healing
- Evaluation for wound infection or mediastinitis
- Assessment of postoperative pain and mobility

Patients commonly require:

- Early postoperative visits within 1–2 weeks
- Additional follow-up during the first several months

2. Minimally invasive coronary bypass surgery
   Potential advantages:

- Smaller incisions
- Reduced wound-related complications
- Faster recovery

As a result:

- Some patients may require fewer wound-related follow-up visits.
- Recovery may progress more rapidly.

However:

- Regular cardiology follow-up remains essential in both approaches.
- Monitoring of graft function, cardiac symptoms, medications, and risk-factor control is necessary regardless of the surgical technique.

In summary, minimally invasive surgery may reduce the need for wound-related follow-up visits because of faster recovery, although long-term cardiac follow-up remains important after both procedures.

40. In the long term, is the need for repeat stenting or a second bypass surgery lower with the minimally invasive approach compared with the conventional method?

The long-term need for repeat stenting or repeat bypass surgery depends primarily on graft durability, progression of coronary artery disease, and patient-related cardiovascular risk factors rather than on whether the procedure was minimally invasive or conventional.

1. Important determinants of long-term outcomes

- Type of graft used (arterial vs venous grafts)
- Severity and progression of coronary artery disease
- Smoking, diabetes, hypertension, and cholesterol control
- Medication adherence and lifestyle modification

2. Minimally invasive coronary bypass surgery

- Often used in selected patients with limited coronary disease.
- Long-term graft patency may be excellent when internal mammary artery grafts are used.

3. Conventional coronary artery bypass surgery

- Frequently preferred in extensive multivessel disease because it allows more complete revascularization.
- Long-term outcomes are well established.

Current evidence:

- The surgical approach itself does not appear to be the primary determinant of future stenting or repeat surgery.
- Complete and durable revascularization is more important than incision size.

In summary, the long-term need for repeat intervention depends mainly on graft quality, coronary disease progression, and cardiovascular risk-factor control rather than on whether the surgery was minimally invasive or conventional.
